# Chemoenzymatic labeling of DNA methylation patterns for single-molecule epigenetic mapping

**DOI:** 10.1093/nar/gkac460

**Published:** 2022-06-03

**Authors:** Tslil Gabrieli, Yael Michaeli, Sigal Avraham, Dmitry Torchinsky, Sapir Margalit, Leonie Schütz, Matyas Juhasz, Ceyda Coruh, Nissim Arbib, Zhaohui Sunny Zhou, Julie A Law, Elmar Weinhold, Yuval Ebenstein

**Affiliations:** School of Chemistry, Center for Nanoscience and Nanotechnology, Center for Light-Matter Interaction, The Center for Physics and Chemistry of Living Systems, Raymond and Beverly Sackler Faculty of Exact Sciences, Tel Aviv University, Tel Aviv, Israel; School of Chemistry, Center for Nanoscience and Nanotechnology, Center for Light-Matter Interaction, The Center for Physics and Chemistry of Living Systems, Raymond and Beverly Sackler Faculty of Exact Sciences, Tel Aviv University, Tel Aviv, Israel; School of Chemistry, Center for Nanoscience and Nanotechnology, Center for Light-Matter Interaction, The Center for Physics and Chemistry of Living Systems, Raymond and Beverly Sackler Faculty of Exact Sciences, Tel Aviv University, Tel Aviv, Israel; School of Chemistry, Center for Nanoscience and Nanotechnology, Center for Light-Matter Interaction, The Center for Physics and Chemistry of Living Systems, Raymond and Beverly Sackler Faculty of Exact Sciences, Tel Aviv University, Tel Aviv, Israel; School of Chemistry, Center for Nanoscience and Nanotechnology, Center for Light-Matter Interaction, The Center for Physics and Chemistry of Living Systems, Raymond and Beverly Sackler Faculty of Exact Sciences, Tel Aviv University, Tel Aviv, Israel; Institute of Organic Chemistry, RWTH Aachen University, D-52056Aachen, Germany; Institute of Organic Chemistry, RWTH Aachen University, D-52056Aachen, Germany; Plant Molecular and Cellular Biology Laboratory, Salk Institute for Biological Studies, La Jolla, CA, USA; Department of Obstetrics and Gynecology, Meir Hospital, Kfar Saba, Israel & Sackler Faculty of Medicine, Tel Aviv University, Tel Aviv, Israel; Department of Chemistry and Chemical Biology, and Barnett Institute of Chemical and Biological Analysis, Northeastern University, Boston, Massachusetts02115, USA; Plant Molecular and Cellular Biology Laboratory, Salk Institute for Biological Studies, La Jolla, CA, USA; Institute of Organic Chemistry, RWTH Aachen University, D-52056Aachen, Germany; School of Chemistry, Center for Nanoscience and Nanotechnology, Center for Light-Matter Interaction, The Center for Physics and Chemistry of Living Systems, Raymond and Beverly Sackler Faculty of Exact Sciences, Tel Aviv University, Tel Aviv, Israel

## Abstract

DNA methylation, specifically, methylation of cytosine (C) nucleotides at the 5-carbon position (5-mC), is the most studied and significant epigenetic modification. Here we developed a chemoenzymatic procedure to fluorescently label non-methylated cytosines in CpG context, allowing epigenetic profiling of single DNA molecules spanning hundreds of thousands of base pairs. We used a CpG methyltransferase with a synthetic *S-*adenosyl-l-methionine cofactor analog to transfer an azide to cytosines instead of the natural methyl group. A fluorophore was then clicked onto the DNA, reporting on the amount and position of non-methylated CpGs. We found that labeling efficiency was increased up to 2-fold by the addition of a nucleosidase, presumably by degrading the inactive by-product of the cofactor after labeling, preventing its inhibitory effect. We used the method to determine the decline in global DNA methylation in a chronic lymphocytic leukemia patient and then performed whole-genome methylation mapping of the model plant *Arabidopsis thaliana*. Our genome maps show high concordance with published bisulfite sequencing methylation maps. Although mapping resolution is limited by optical detection to 500–1000 bp, the labeled DNA molecules produced by this approach are hundreds of thousands of base pairs long, allowing access to long repetitive and structurally variable genomic regions.

## INTRODUCTION

DNA methylation is an epigenetic mark that plays a major regulatory role in transcription, gene regulation, and disease. 5-methylcytosine (5-mC) is conserved among plants and mammals, and its precise genomic patterns are crucial for development. In mammals, 5-mC occurs most often in the context of CpG dinucleotides and, in the human genome, most CpGs are highly methylated (between 70 and 80%). However, in specific locations, mainly high-density CpG islands (CGI), they remain mostly non-methylated. The majority of CGIs (∼70%) are located at gene promoters and the methylation status of these regions is known to regulate gene expression (1–3). In cancer, large-scale changes in methylation levels are observed and can include both genome-wide hypomethylation as well as more localized hypermethylation, mainly of tumor suppressor genes that are often silenced in cancer (4–8).

Methylation is established by a diverse family of methyltransferases (MTases). These enzymes utilize *S*-adenosyl-l-methionine (AdoMet) as the methyl donor, forming methylated DNA and *S*-adenosyl-l-homocysteine (AdoHcy) as a by-product (9,10). Various approaches have been developed to specifically profile cytosine DNA methylation, both on a global level and at base-pair resolution. The gold standard technique is bisulfite sequencing (called BS-seq (11)) or methylC-seq (12) depending on the methods used), which relies on chemical conversion of unmodified cytosines to uracil, while leaving methylated cytosines unconverted (11,12). After PCR amplification and sequencing, the originally methylated cytosines are read as C while the unmodified cytosines are read as T. However, this method can suffer from several limitations, such as high DNA degradation and potential biases due to the amplification process. Short read sequencing also adds several drawbacks and mainly the limitation in characterizing large variable and repetitive regions, as well as population averaging of the data which masks cell-to-cell variation (13). Single-cell bisulfite sequencing may profile individual methylomes (14,15) but is still limited in characterizing large variable and repetitive regions.

Third-generation sequencing approaches, including single-molecule, real-time (SMRT) sequencing (Pacific Biosciences Inc.), and nanopore sequencing (Oxford Nanopore Technologies Ltd.), are able to detect methylated cytosines directly (16–20). However, whole-methylome analysis on these platforms is still challenging and costly (21).

Whole-genome epigenetic profiling by optical genome mapping (Bionano Genomics Inc.) is a new addition to the epigenetic mapping toolbox. This technology is based on stretching long genomic DNA fragments for optical imaging. The DNA is labeled with two colors, one is used for aligning the molecules to the reference genome, and the other reports on the epigenetic content of the molecules (22–24). This technique outputs extremely long, single-molecule data (N50–200 kb) that allows profiling of large variable and repetitive regions (25).

Our group developed Reduced representation Optical Methylation mapping (ROM), as a method for the labeling and detection of non-methylated CpG sites using the bacterial MTase, M.TaqI, and a cofactor analogue (23). The non-methylation labeling scheme relies on the transfer of a fluorescent molecule from a synthetically modified form of the native methylation cofactor AdoMet. The enzyme is ‘tricked’ into transferring the extended chemical group with a fluorophore instead of the natural methyl group, resulting in fluorescence at non-methylated CpG sites. The long-read methylation data allowed us to assemble high coverage human methylomes and analyze the methylation status of promoters and their distal enhancers, simultaneously imaged on the same long DNA molecules (26). However, M.TaqI (recognition site: TCGA) only samples about 6% of human CpG sites and thus can fail to capture vital epigenetic information.

**Figure 1.**
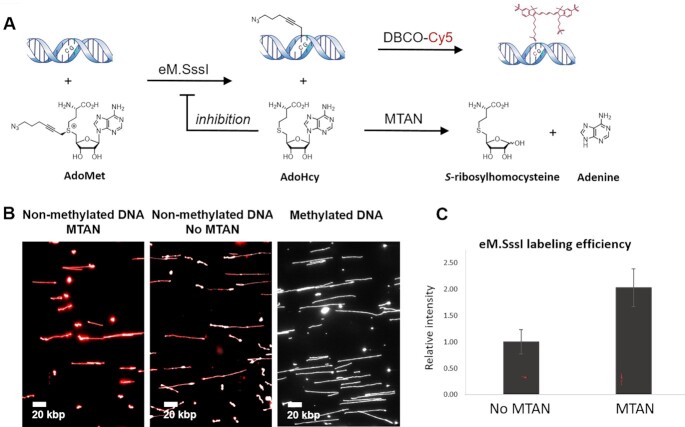
(**A**) eM.SssI catalyzes the transfer of an azide from AdoYnAzide to non-methylated CpGs. DBCO-cy5 is then attached by click chemistry to the azide-modified CpGs. Following the transfer of the azide-containing side chain by eM.SssI, the remaining cofactor by-product AdoHcy serves as a substrate for MTAN that hydrolyzes it to adenine and *S*-ribosylhomocysteine. In the absence of MTAN, AdoHcy accumulates and can bind to eM.SssI, inhibiting its activity. (**B**) A representative field of view of non-methylated λ DNA labeled with eM.SssI, in the presence (left) or absence (middle) of MTAN. Methylated DNA was labeled as control (right). In gray-DNA backbone, stained by YOYO-1, showing the DNA molecule contour. In red- non-methylated CpG labels. (**C**) Global quantification (calculated by dividing the total intensity of the CpG label by the total length of the DNA) of non-methylated CpGs labelled with eM.SssI, with or without MTAN.

Here, we present a chemoenzymatic labeling approach for the detection of all non-methylated CpGs as a means for global methylation quantification and for genomic methylation mapping. This is achieved by utilizing the CpG-specific MTase M.SssI from the bacteria *Spiroplasma* sp. strain MQ-1 that naturally transfers a methyl group to the fifth position of cytosines in CpG dinucleotides (27,28). While M.SssI has the potential to label all non-methylated CpGs, it is not able to utilize modified AdoMet cofactors. However, an engineered variant of M.SssI (Q142A/N370A) is able to transfer an azide group to non-methylated CpG sites by processing an azide-modified cofactor AdoYnAzide (Ado-6-azide). This engineered M.SssI (eM.SssI) was used by Kriukiene *et al.* for non-methylation sequencing (29). However, the affinity capture on streptavidin-modified beads showed a 20–30% capture efficiency, indicating poor labeling yield which may prove problematic for single-molecule optical mapping. To increase labeling efficiency, we employed the enzyme 5-methylthioadenosine/*S*-adenosylhomocysteine nucleosidase (MTAN, EC 3.2.2.9) that catalyzes the hydrolysis of the glycosidic bond in AdoHcy. Thus, MTAN degrades the inactive cofactor by-product, AdoHcy, formed from the natural cofactor AdoMet or synthetic cofactor analogues, like AdoYnAzide (Figure 1A). In general, AdoHcy is a well-known product inhibitor for MTases (30). Lowering its concentration by the addition of MTAN effectively drives the reaction towards increased labeling efficiency (Figure 1A). The azide-modified DNA can then be covalently labeled by a dibenzocyclooctyne-cy5 (DBCO-cy5) fluorophore in a strain-promoted azide-alkyne cycloaddition (SPAAC, copper-free click chemistry reaction), to specifically label non-methylated CpG sites. Thus, the increased efficiency of the eM.SssI/MTAN combination was used to generate optical maps that present the genome-wide epigenetic status of CpG sites with single-molecule sensitivity.

Here, we apply this labeling strategy to human peripheral blood mononuclear cells (PBMCs), emphasizing the ability to quantify the methylation status of CpG sites in healthy vs. cancer patients with high sensitivity. We further demonstrate whole-genome methylation mapping of the model plant *A. thaliana*. Our approach allows simultaneous genetic and epigenetic characterization of long stretches of DNA at the single-molecule level, and potentially permits studying variations between single cells (26).

## MATERIALS AND METHODS

### Human subjects

The healthy donor sample used in this study was collected with informed consent for research use and approved by the Tel-Aviv University and Meir medical center ethical Review Boards, in accordance with the declaration of Helsinki. The PBMCs from a CLL donor were purchased from BioServe.

### High-molecular-weight DNA extraction

#### Plants

High molecular weight (HMW) DNA was extracted for optical mapping from 1 g of 6–8 weeks old whole plant tissue, before flowering using the BioNano Genomics Fix’n’Chop protocol with some modifications. Briefly, no formaldehyde was used, chopping was done only with a lab blender and no razor blade was used, 7.5% of 2-mercaptoethanol was used. Following the final wash, the nuclei pellet was resuspended in cell suspension buffer (CHEF mammalian DNA extraction kit, Bio-Rad) and incubated at 43°C for 10 min. 2% low melting agarose (CleanCut agarose, Bio-Rad) was melted at 70°C followed by incubation at 43°C for 10 min. Melted agarose was added to the resuspended cells at a final concentration of 0.75% and mixed gently. The mixture was immediately cast to a plug mold and plugs were incubated at 4°C until solidified.

#### Human subjects

For human peripheral blood mononuclear cells (PBMCs), peripheral blood of a healthy donor was isolated by density gradient centrifugation using Ficoll Paque Plus (GE Healthcare) according to manufacturer's instructions. Plugs were prepared according to Plug Lysis protocol (BioNano Genomics). CLL sample was purchased as separated PBMCs from a CLL patient and was thawed at 37°C water bath immediately prior to use. For both healthy and CLL samples, 1 × 10^6^ PBMCs were washed twice with PBS and mixed with 2% low melting agarose as described above.

All plugs, from human or plant origin, were incubated twice (2 h incubation followed by an overnight incubation) at 50°C with freshly prepared 167 μl Proteinase K (Qiagen) in 2.5 ml lysis buffer (BioNano Genomics) with occasional shaking. Next, plugs were incubated with 50 μl RNase (Qiagen) in 2.5 ml TE (pH 8) for 1 hour at 37°C with occasional shaking. Plugs were washed three times by adding 10 ml wash buffer (10 mM Tris, pH 8, 50 mM EDTA), manually shaking for 10 seconds, and discarding the wash buffer before adding the next wash. Plugs were then washed four times by adding 10 ml wash buffer and shaking for 15 min on a horizontal platform mixer at 180 rpm at room temperature. Following washes, plugs were stored at 4°C in wash buffer or used for labeling. In order to extract high molecular weight DNA, plugs washed three times in TE pH 8 with shaking on a horizontal platform as explained, were melted for 2 min at 70°C, followed by 5 min incubation at 43°C. Next, 0.4 units of Gelase (Thermo Fisher) were added and the mixture was incubated for 45 min. Viscous DNA was gently pipetted and incubated at room temperature overnight in order to achieve homogeneity. DNA concentration was determined using Qubit BR dsDNS assay.

### Genetic barcoding and non-methylated CpG labeling

Genetic barcodes were produced by nick translation, a modified version of Irys prep NLRS protocol (Bionano Genomics). Briefly, 300 ng DNA were nicked with 10 units of Nt.BspQI (NEB) at specific sequence motifs (GCTCTTCN^) in the presence of 1 μl 10× buffer 3.1 (NEB) for 2 h at 50°C and ultra-pure water to a volume of 10 μl. For DNA labeling, 15 units of Taq DNA polymerase were incubated with 200 nM of dATP dGTP dCTP (Sigma) and atto-532-dUTP (Jena Bioscience) in the presence of 1.5 μl of 10× thermopol buffer and ultra-pure water to a volume of 15 μl, the reaction was incubated at 72°C for 1 h. Nicks were then ligated for 30 min at 37°C using 4 units of Taq DNA ligase (NEB) in the presence of 0.5 μl thermopol buffer, 20 μM dNTPs (Sigma) 1 mM NAD+ (NEB) and water to a volume of 20 μl. For non-methylated CpG labeling (mTAG labeling) 300 ng of nick-labeled DNA were incubated overnight at 37°C with 3.2 μM of the CpG-specific DNA cytosine-C5 MTase M.SssI double mutant Q142A/N370A (eM.SssI) (29) in the presence of 80 μM cofactor analogue AdoYnAzide (31) and 3 μl 10X M.SssI reaction buffer (Thermo Fisher/10 mM Tris–HCl, 50 mM NaCl, 1 mM DTT, pH 7.9), in a total reaction volume of 30 μl. The reaction was supplemented with 2 μM 5′-methylthioadenosine/*S*-adenosylhomocysteine nucleosidase (MTAN) and after 2 h of incubation, a spike-in of 0.8 μM eM.SssI and 2 μM MTAN (32, 33) was added to the reaction to further improve labeling efficiency. Next, 60 μg of Proteinase K (PK) were added and incubated for 2 h at 45°C. Following PK digestion, 700 μM of dibenzocyclooctyne-cy5 (DBCO-cy5) were added and incubated overnight at 37°C. The Dual-labeled DNA was then embedded in low melting agarose gel plugs and washed in 10 ml wash buffer (20 mM Tris–HCl, 50 mM EDTA, pH 8) for 15 min 5 times and twice with TE pH 8. Plugs were melted at 70°C for 2 min and incubated at 43°C for 5 min. 2 μl of beta-agarase (Thermo Fisher Scientific) were added for agarose digestion and the reaction was incubated at 43°C for 45 min. Dual-labeled DNA was stained with DNA stain (BioNano Genomics) according to the Irys Prep NLRS protocol with the addition of 25 mM Tris pH 8 and 30 mM NaCl. DNA concentration was measured by Qubit HS dsDNA assay.

To test non-methylation labeling efficiency, DNA was labelled with eM.SssI as described above, with or without MTAN. All DNA samples were purified from excess fluorophores, then applied on a custom epoxy-covered multi-well slide and imaged using a commercial slide scanner, as described in the work of Margalit *et al.* (34).

### Modification-restriction assay

Twofold serial DNA MTase dilutions (20 μl) were obtained by preparing a solution (40 μl) of eM.SssI (220 ng/μl, 4.86 μM) in reaction buffer (10 mM Tris–HCl, 50 mM NaCl, 1 mM DTT, pH 7.9) containing phage λ DNA (50 ng/μl, 1.56 nM, 3112 CpG sites, 4.86 μM CpG sites) and AdoYnAzide (80 μM). An aliquot of this solution (20 μl) was removed and added to reaction buffer (20 μl) containing λ DNA (50 ng/μl) and cofactor (80 μM) to give a 2-fold eM.SssI dilution. This step was repeated several times to yield 4-, 8-, 16-fold etc. eM.SssI dilutions with 1, 0.5, 0.25, 0.125, 0.0625 etc. equivalents of eM.SssI per CpG site. eM.SssI dilutions containing MTAN (2 μM) were prepared accordingly. The reaction mixtures were incubated at 37°C for 1 h, heat inactivated at 65°C for 20 min, supplemented with restriction endonuclease R.HhaI (30 μl, 10 units in 20 mM Tris-OAc, 10 mM Mg(OAc)_2_, 50 mM KOAc, 0.1 mg/ml BSA, pH 7.9) and incubation was continued at 37°C for 1 h. The reaction mixtures were treated with proteinase K (1 μl, 20 μg/μl), incubated at 53°C for 30 min and 6× loading buffer (10 μl, 0.25% bromphenol blue, 30% glycerol) was added. Samples (6 μl) were analyzed by agarose gel (1%) electrophoresis in the presence of GelRed (0.1 μl stock solution/ml gel). DNA bands were visualized with a UV transilluminator (312 nm) and documented with a CCD camera equipped with a filter (540 ± 50 nm).

### HPLC efficiency assay

Transfer of azide groups (1. step): Solutions (200 μl) of eM.SssI (113 ng/μl, 2.5 μM) in reaction buffer (10 mM Tris–HCl, 50 mM NaCl, 1 mM DTT, pH 7.9) containing duplex oligodeoxynucleotide (ODN) (31) (hybridization of 5′-ATT ATT ATT ATT AG**C G**CA TTA TTA-3′ and 5′-TAA TAA TG**C G**CT AAT AAT AAT AAT-3′, 36.7 ng/μl, 2.5 μM, 2.5 μM CpG sites) and AdoYnAzide (80 μM) were prepared without MTAN and with MTAN (2 μM). The reaction mixtures were incubated at 37°C for 1 h. Proteinase K (6 μl, 20 μg/μl) was added to both reaction mixtures and incubation continued at 45°C for 2 h, followed by heat inactivation at 80°C for 3 h.

Fluorescence labeling (2. step): Half of each reaction mixture (100 μl) was supplemented with DBCO-Sulfo-Cy5 in DMSO (7.5 μl, 10 mM) to give final DBCO-Sulfo-Cy5 concentrations of 700 μM. The click reactions mixtures were incubated at 37°C overnight.

All reaction mixtures were purified using NAP-5 columns following the instructions of the manufacturer. Equilibration (10 ml) and elution (500 μl) was done with nuclease P1 buffer (0.2 mM ZnSO_4_, 20 mM NaOAc, pH 5.3). Purified duplex ODN were supplemented with nuclease P1 (3 μl, 10 U/μl) and incubated at 37°C for 2 h. CIAP (1.5 μl, 0.6 U/μl), CIAP buffer (60 μl, 500 mM Tris–HCl, 10 mM MgCl_2_, pH 9.0) and deionized water were added to a total volume of 600 μl and samples incubated at 37°C overnight. Nucleosides were separated by reverse-phase HPLC (Prontosil C-18 AQ column, 250 × 4.6 mm, 5 μm, 120 Å, equipped with a C-18 precolumn, 8.0 mm × 4.0 mm, 5 μm, 120 Å) using an acetonitrile gradient (3.5–5.7% in 20 min and 5.7–70% in 22 min) in aqueous triethylammonium acetate buffer (10 mM, pH 7.0) with a flow of 1 ml/min and detection at 260 nm/272 nm or 260 nm/646 nm for runs with Cy5-labeled nucleosides.

Peaks of the nucleosides at 260 nm were integrated using Empower 2 software and areas were normalized by dividing them by the respective extinction coefficients at 260 nm (dC: 7009 mol l^–1^ cm^–1^; dG: 11715 mol l^–1^ cm^–1^; dT: 8902 mol l^–1^ cm^–1^; dA: 15663 mol l^–1^ cm^–1^; 5-azido-dC = 5mdC: 5435 mol l^–1^ cm^–1^). Normalized areas of dT were divided by the number of dT residues in the duplex ODN (20 residues) to obtain a normalized area for one nucleoside. Normalized areas for the other nucleosides were divided by this value to give experimental amounts for all nucleosides (see Supplementary Table S1). Yields for 5-azido-dC were calculated by dividing the amount of 5-azido-dC by the sum of dC and 5-azido-dC multiplied by 2 (four dC/two target dC). Yields for 5-Cy5-dC were calculated from the decrease of 5-azido-dC.

### Glass slide preparation and quality-control imaging

22 × 22 mm^2^ glass cover-slips were cleaned for at least 7 hours to overnight by incubation in a freshly made 2:1 (v/v) mixture of 70% nitric acid and 37% hydrochloric acid. After extensive washing with ultrapure water (18 MΩ) and then with ethanol, coverslips were dried under a stream of nitrogen. Dry cover-slips were immersed in a premixed solution containing 750 μl *N*-trimethoxysilylpropyl-*N*,*N*,*N*-trimethylammonium chloride and 200 μl of vinyltrimethoxysilane in 300 ml ultrapure water and incubated overnight at 65°C. After incubation, cover-slips were thoroughly washed with ultrapure water and ethanol and stored at 4°C in ethanol. The silane solution was freshly made and thoroughly mixed before the cover-slips were introduced into the mixture. Stored cover-slips were normally used within 2 weeks.

For quality control, samples were imaged to evaluate DNA length and degree of labeling. Samples were diluted 1:100–1:300 in TE with DTT buffer (10 mM Tris pH 8, 1mM EDTA, 200 mM DTT, Sigma) and were stained with 130 nM YOYO-1 (Invitrogen) DNA intercalating dye. DNA molecules were stretched by placing 8 μl of the solution at the interface of an activated coverslip placed on a standard microscope slide. The extended DNA molecules were imaged with a fluorescence microscope (TILL Photonics GmbH) using an Olympus UPlanApo 100× 1.3 NA oil immersion objective. Each image was composed of three colors, the YOYO-1, atto-532 and the Cy5 fluorophores, and was therefore imaged with the appropriate filters (485/20, 537/26 and 650/13 bandpass excitation filters, 525/30, 578/16 and 684/24 bandpass emission filters, for YOYO-1 and Cy5, respectively). Images were acquired by a DU888 EMCCD (Andor technologies) with an EM gain setting of 300 and exposure times of 100 for YOYO-1 and 2000 ms for atto-532 and Cy5.

### Global methylation quantification

Differences in global fluorescence intensity following labeling with and without MTAN, and between genomic samples were analyzed by an in house software (35,36) (https://github.com/ebensteinLab/Tiff16_Analyzer). The code measures the total length of each molecule as well as the number and intensity of CpG labels detected along the DNA molecules. Quantification of non-methylated CpG was done by dividing the total intensity of the CpG label by the total length of the DNA (calculated as intensity of methylation signal/total DNA length in kbp). Similar analysis was performed on images of DNA stretched in nanochannels from CLL patient and healthy donor. In total we sampled 5.585 Gb from CLL patient and 330 Mb from the healthy donor and a total of 9840 field of view (FOV) from each donor. The two samples have a ∼17-fold difference in the amount of sampled DNA and therefore we have confirmed that this fact does not create a bias in the interpretation of the results. A running average was calculated for each sample by random selection of molecules from the dataset (Supplementary Figure S7). We confirm that the average methylation level remains stable even when sampling only 100 Mbp of genomic DNA, and therefore both samples were sufficiently oversampled to provide reliable global methylation levels.

### Optical mapping and analysis

Loading of DNA in nanochannels and imaging were performed using an Irys instrument (BioNano Genomics). Detection of imaged molecules and fluorescent labels along each molecule was performed by AutoDetect (version 2.1.4, BioNano Genomics). Alignment to the reference genome was performed using IrysView software (version 2.3, BioNano Genomics). *Arabidopsis* accession col-0 was run on a single chip on the Irys platform (BioNano Genomics), for up to 120 cycles to collect up to 89 Gb of quality filtered data. Molecule files (.bnx) were loaded into IrysView, quality filtered (>100 kb length, >2.75 signal/noise ratio) and aligned to the reference TAIR10 (https://www.arabidopsis.org/download/index-auto.jsp?dir=%2Fdownload_files%2FGenes%2FTAIR10_genome_release). Reference genome was converted into .cmap format and used to align the detected molecules using standardized parameters (-nosplit 2 -BestRef 1 -biaswt 0 -Mfast 0 -FP 1.5 -FN 0.15 -sf 0.2 -sd 0.0 -A 5 -outlier 1e-3 -outlierMax 40 -endoutlier 1e-4 -S -1000 -sr 0.03 -se 0.2 -MaxSF 0.25 -MaxSE 0.5 -resbias 4 64 -maxmem 64 -M 3 3 -minlen 100 -T 1e-6 -maxthreads 32 -hashgen 5 3 2.4 1.5 0.05 5.0 1 1 3 -hash -hashdelta 10 -hashoffset 1 -hashmaxmem 64 -insertThreads 4 -maptype 0 -PVres 2 -PVendoutlier -AlignRes 2.0 -rres 0.9 -resEstimate -ScanScaling 2 -RepeatMask 5 0.01 -RepeatRec 0.7 0.6 1.4 -maxEnd 50 -stdout -stderr –usecolor 1). Alignments were analyzed for genome coverage, and gaps using the .xmap output file.

Following alignment, data was uploaded to IrysExtract (37), (https://www.nanobiophotonix.sites.tau.ac.il) to generate an average intensity profile of fluorescence intensity across the genome. We used all molecules aligned with a confidence equal or higher than 8 (*P* ≤ 10^–8^) and that had at least 70% of each molecule aligned to the reference. All regions in the genome with less than 15× coverage (3% of the genome), were excluded from the data. The analysis pipeline results in a genome-wide intensity bedgraph file at 500 bp resolution. The file allows further analysis and visualization in a genome browser as seen in Figures 4 and 5.

### Comparison of optical methylation mapping to bisulfite sequencing

Genome-wide bisulfite sequencing data (GSE43857) (38) was processed to generate a bedgraph track of non-methylated CpGs represented as a “methylation score.” To calculate the methylation score, the non-methylated CpGs were padded ±500 bp and summed over 1 kb bins to generate the bedgraph file. The score in each bin was then divided by the maximum score, setting the data between 0–1. The genome was then divided to 10 kb windows and the mean score from each method (methylation score for the bisulfite data and relative intensity for the optical mapping data) was calculated for each window, K–S test was performed to test normality and since the data do not normally distribute, Spearman correlation analysis was performed using SPSS (IBM Corp. Released 2016. IBM SPSS Statistics for Windows, Version 24.0), to determine the linear dependence between bisulfite sequencing and methylation mapping.

### Analysis of CpG methylation distribution across gene body and siRNA

*pol-iv*-dependent 24 nt short interfering RNAs (siRNA) was retrieved from the previously published data (39). miRNA primary transcript loci were extracted from miRBase ath.gff3 file (http://www.mirbase.org/ftp.shtml). *Arabidopsis* TAIR10 TAS loci were obtained from the ta-siRNA database (40). The bed files of the 24nt siRNA loci, miRNA precursor loci, and TAS loci were used to measure the non-methylated CpGs from optical mapping and genome-wide bisulfite sequencing as detailed below (Supplementary File 5).

First, a file containing all CpGs was created from TAIR10 FASTA by knickers (version 1.5.5 Bionano Genomics). Then, a non-methylated CpG track from genome-wide bisulfite sequencing data of *A. thaliana* was created using BEDTools (version 2.25.0) subtract (41). Each site in the non-methylated CpG track was expanded by 500 bp on both sides to adjust to the optical mapping resolution. Genome coverage of non-methylated CpG was generated by BEDTools genomecov to receive a non-methylated CpG bedgraph. Finally, this file was uploaded to deepTools (42) in addition to the average label intensity from optical mapping and the clusters bed file. Using computeMatrix in reference point mode, we computed the mean value for each bin from all regions 10 kb upstream and downstream from the clusters. All clusters were set to position 0, and median coverage for the neighboring regions in 1 kb bins was plotted.

For gene body analysis, we compared the average label intensity of non-methylated CpGs from optical mapping and genome-wide bisulfite sequencing data with a bed file containing all *A. thaliana* genes (TAIR10) (supplementary file 6). Using computeMatrix in scale-region mode, all genes were scaled to the same length in 100 bp bins. We added 1500 bp upstream and downstream to the TSS and TES (respectively) and computed the mean value for each bin.

## RESULTS AND DISCUSSION

### Efficient labeling of non-methylated CpGs

CpG methylation is a binary state and on the single-molecule level a site can be either methylated or non methylated. Neverthless, methylation level is reported on a scale of 0–1 to account for the average methylation of a CpG locus in a population of cells. In order to accurately report on such average population levels, the methylation status of each CpG must be sampled over multiple DNA molecules containing this locus. Single-molecule epigenomic mapping requires highly efficient labeling in order to extract reliable information from the studied sample. We hypothesized that during DNA alkylation by eM.SssI, the cofactor by-product AdoHcy could reduce the overall DNA alkylation efficiency by occupying the cofactor binding pocket and preventing additional alkylation cycles (Figure 1A). Thus, we sought to enzymatically degrade this by-product by the addition of recombinant MTAN (43). To test the effect of MTAN on the overall labeling efficiency, we labeled non-methylated λ DNA using eM.SssI, AdoYnAzide, and DBCO-cy5, with or without MTAN (Figure 1B and Supplementary Figure S1). Figure 1C shows the relative fluorescence intensity of λ DNA labeled with or without MTAN. Average labeling intensity increased two-fold with the addition of MTAN to the reaction (Figure 1C, Supplementary Figure S2). The relative labeling efficiency was further tested and quantified with and without MTAN by a multi-well slide fluorescence assay (Supplementary Figure S3) as well as a modification-restriction assay (Supplementary Figure S4) which showed a similar improvement in labeling in the presence of MTAN. A quantitative HPLC efficiency assay could only be performed on a short duplex DNA. In our case, a 24 bp ODN containing one CpG site on each strand (Supplementary Figure S5, Supplementary Table S1). The alkylation efficiency of the short duplex was measured to be 67% and increased to 80% with addition of MTAN. The high initial alkylation efficiency could result from the higher modification yields with short duplex DNA compared to native genomic DNA leading to a smaller increase with MTAN. We note that the short ODN contains much higher density of labeling sites (1 to 24 bp in the ODN vs. 1 to ∼150 bp for genomic DNA).

Since the optical resolution (∼500–1000 bp) is lower than the density of CpG sites in CpG-rich regions in the genome, we verified that the fluorescence intensity increases linearily with the increase in labeled CpG sites (Supplementary Figure S6). Thus, although a single fluorescent spot may contain several CpG sites that cannot be resolved, the intensity of the spot reports on the density of labeled CpG sites.

### Global methylation quantification

**Figure 2.**
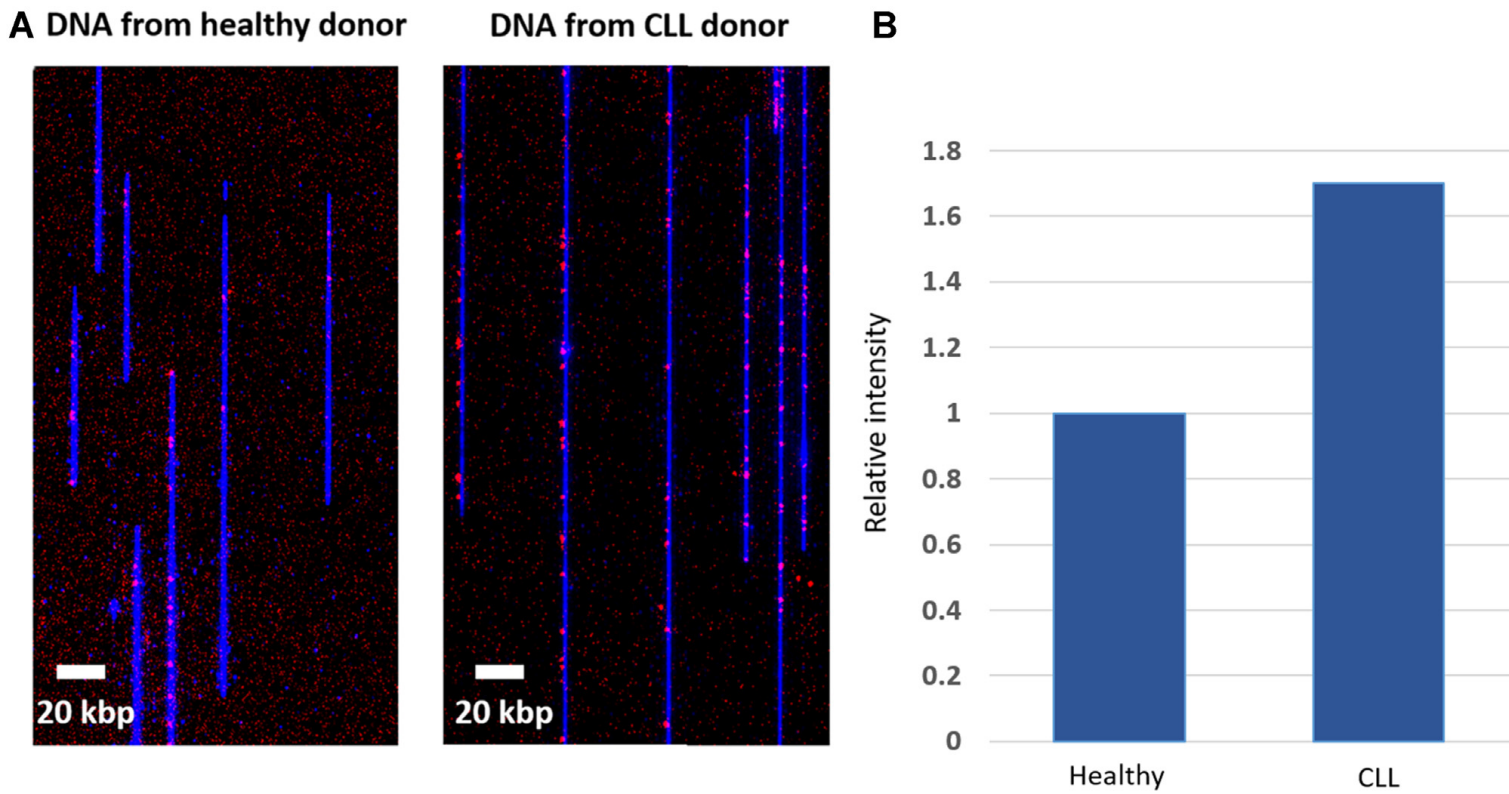
Methylation quantification in human peripheral blood mononuclear cells (PBMCs) from one healthy and one CLL patient donor. (**A**) Representative images of stretched DNA in nanochannel array chips from both samples: DNA backbone in blue and epigenetic labels in red (more red signal denotes less DNA methylation at CpG sites). (**B**) Global quantification of the methylation signal intensity along the DNA molecules. Intensity of the healthy sample was normalized to 1.

One of the hallmarks of cancer is a global reduction in genomic methylation levels (44). As a first step in evaluating our optical mapping technique for epigenetic profiling, we quantified the genome-wide methylation level of PBMCs from a single patient diagnosed with chronic lymphocytic leukemia (CLL) and a healthy donor. Both healthy and patient DNA was stretched in nanochannel array chips, imaged, and the fluorescence from the non-methylated CpG labels was detected (Figure 2A). Fluorescence intensity along the labeled DNA molecules was automatically measured to determine the relative difference in labeling (36). The relative global methylation level was measured by summing the overall red intensity along the DNA molecules (i.e. non-methylated cytosines in CpG context), normalized to the total DNA length measured for each sample (Figure 2B). When comparing genomic DNA from a patient and a healthy donor, the cancer cells show a global reduction in methylation levels indicated by a higher fluorescence signal along the DNA (Figure 2B). Specifically, the signal intensity from non-methylated CpGs increased by 1.7-fold in the CLL sample compared to the healthy control, indicating global hypomethylation in the CLL sample as previously reported (Supplementary Figure S7, Supplementary Files 2 and 3) (45). Incorporation of our novel chemoenzymatic labeling method into single-molecule optical mapping allows us to distinguish global DNA methylation differences between samples, opening up the potential application of this technique for DNA methylation research and medical diagnostics.

### Genomic mapping of DNA methylation

**Figure 3.**
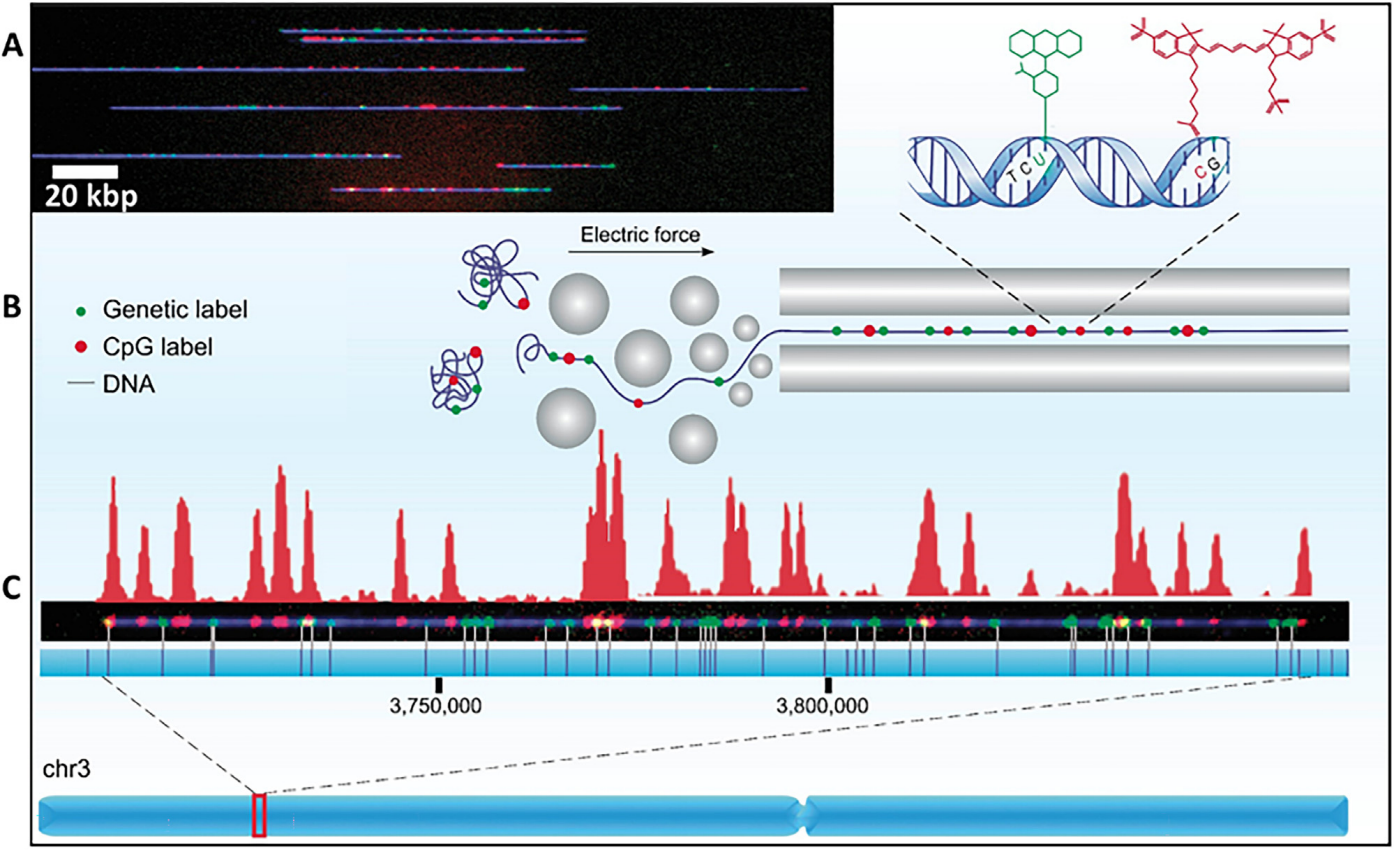
Optical methylation mapping scheme. (**A**) A representative image of stretched DNA molecules in a nanochannel array. DNA backbone in blue (YOYO-1), genetic labels in green (Atto-532) and non-methylated CpG labels in red (cy5). (**B**) Schematic representation of the fluorescently labeled molecules unraveled and extended in a nanochannel array by using an electric field. (**C**) A 150 kb DNA molecule aligned to chromosome 3 by the green genetic labels. The vertical lines on the blue strip represent the theoretical positions of genetic labels in the reference genome. The fluorescence intensity pattern representing the levels of non-methylated CpG sites along the molecule is presented in red above the molecule.

We next attempted to map the CpG methylation profile in the well-characterized genome of the model plant, *Arabidopsis thaliana*. This genome is composed of five chromosomes with a total size of ∼135 Mb. The relatively small genome allowed us to test mapping feasibility while avoiding the cost and computational complexity of analyzing the human genome. In *A. thaliana* DNA methylation commonly occurs in all sequence contexts (CG, CHG and CHH, where H = A, T or C) (10,12) and the presence of methylation at transposons and repeats, as well as some protein-coding genes, is associated with gene silencing and genome stability (12,38,46). *A. thaliana* DNA was extracted, non-methylated CpGs were labeled using our modified M.SssI system, and genomic mapping was performed by using nickase labeling to create a barcode of specific sequence motifs. The dual-labeled DNA molecules were then electro-kinetically forced into nanochannels and imaged on the Bionano Genomics (Inc) Irys system, allowing the detection of fluorescent labeling patterns (Figure 3A and B). We detected 34 866 DNA molecules longer than 100 kb and 28,917 were successfully aligned to the TAIR10 *A. thaliana* reference genome (Supplementary Figure S8). The analysis resulted in an average mapping depth of 47×, covering ∼97% of the genome with a minimum coverage of 15 molecules. The intensity profile of the epigenetic labels for each molecule was created by Irys Extract (37), followed by calculation of normalized average intensity across all molecules aligned to the same region (Figure 3C and Supplementary Figure S9).

### Optical methylation mapping is well correlated with bisulfite sequencing

**Figure 4.**
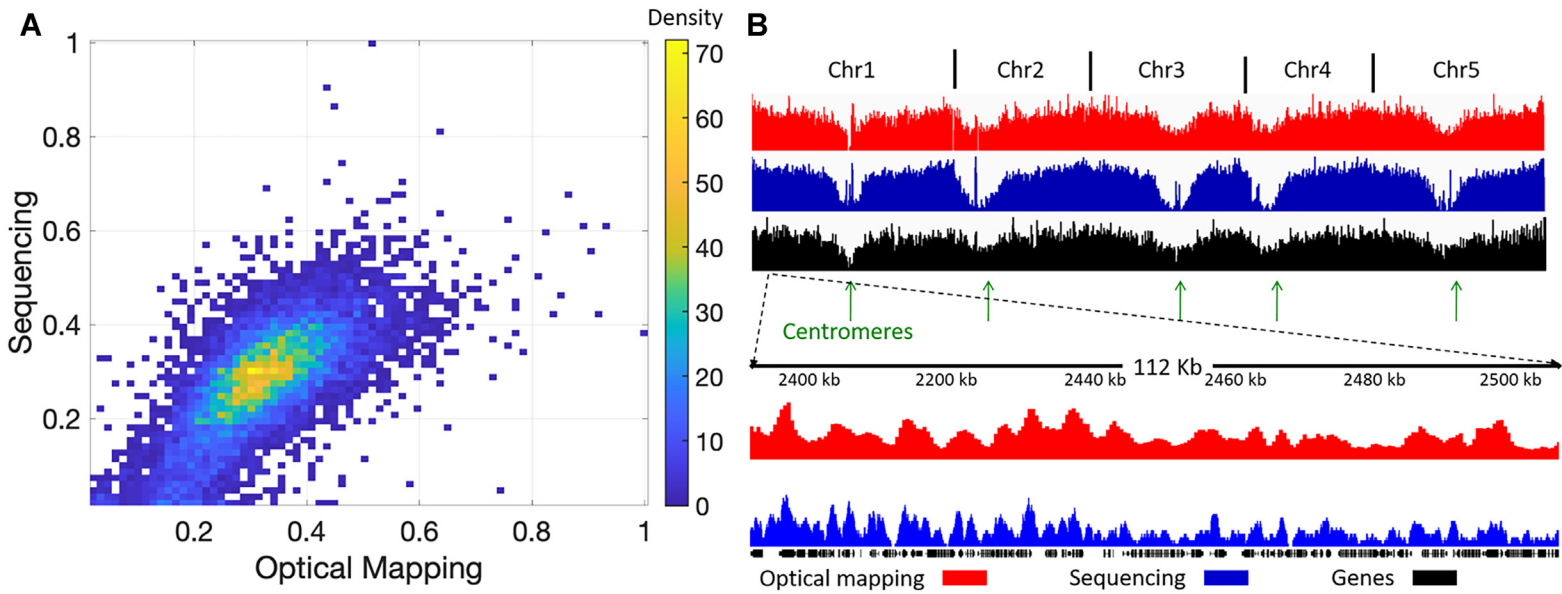
Global correlation between genome-wide bisulfite sequencing and methylation optical mapping. (**A**) Density scatter plot representing the global correlation between genome-wide bisulfite sequencing and optical mapping in 10 kb bins. (**B**) Global view of the optical methylation profiles (relative intensity) in red and the genome-wide bisulfite sequencing data (methylation score) in blue, both showing the levels of non-methylated CpG sites. For reference, the genes are shown in black and the centromeres are indicated by green arrows. The bottom panel presents a zoomed-in view of a selected region.

To further evaluate methylation mapping, a genome-wide correlation of publicly available bisulfite sequencing data (38) with epigenetic optical mapping was calculated. Since our method captures signal from non-methylated CpGs, we present the genome-wide bisulfite sequencing data as the non-methylated fraction of CpGs. To overcome scaling and resolution differences between the two methods, each non-methylated CpG from the bisulfite sequencing data was padded ±500 bp and summed across the genome in 10 kb bins to generate a ‘methylation score’ that corresponds to the amount of non-methylated CpG sites in each bin (i.e. a higher score equates to more non-methylated CpG sites,). This binned data was then compared to the average intensity profile of optical epigenome mapping. First, optical data were normalized for coverage variations by dividing the summed local intensity by the local coverage to obtain a fluorescence intensity score in 1 kb bins across the genome. The normalized intensity score in each bin was then divided by the global maximum intensity (e.g. the 1 kb bin with the highest fluorescence intensity score), setting the relative intensity data between 0–1. As presented in Figure 4A, the optical mapping data nicely correlate with the genome-wide bisulfite sequencing data with a Spearman correlation coefficient of 0.745 (Supplementary File 4). In addition, a global view of the *A. thaliana* genome showing the levels of non-methylated CpG sites illustrates the correlation between the optical mapping and genome-wide bisulfite sequencing data (Figure 4B). The centromeres of the *A. thaliana* genome are enriched in methylated CpGs and are thus seen as dips in the optical graphs at the centromeric positions (low signal denotes high methylation, Figure 4B). The lower panel of Figure 4B shows a zoomed-in region illustrating the positive correlation between the two methods. The finer details in the blue track highlight the higher resolution of the genome-wide bisulfite sequencing data. Nevertheless, the long molecules observed by optical mapping (>100 kb) allowed us to assemble a reliable and highly contiguous epigenetic profile of the *A. thaliana* genome.

### Optical patterns correlate with genome-wide bisulfite sequencing at siRNA clusters and gene bodies

**Figure 5.**
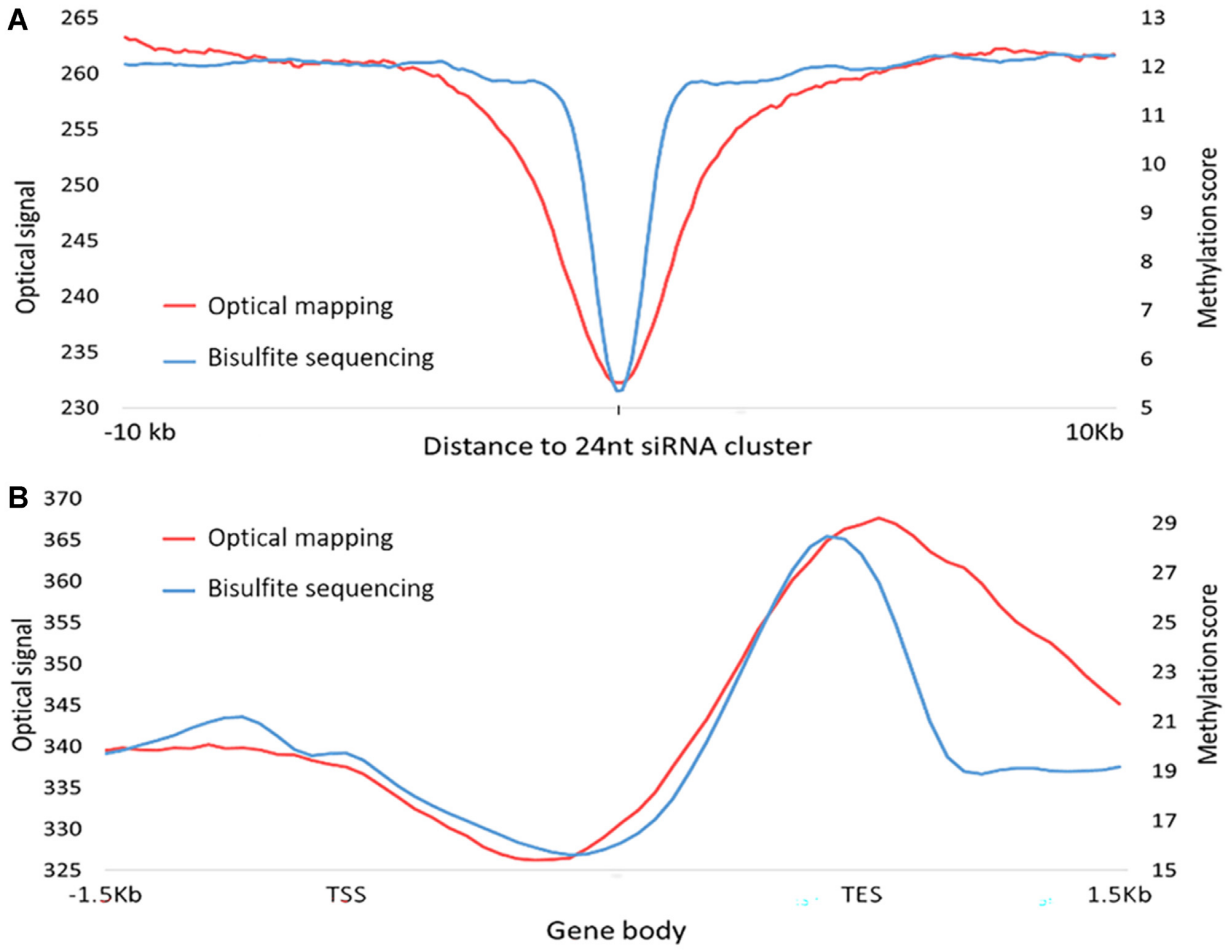
Methylation profiles at 24nt siRNA producing loci and across gene bodies. Genome-wide bisulfite sequencing data in blue & optical mapping data in red. (**A**) 24nt siRNA loci. (**B**) Methylation profile across gene bodies and 1.5 kb upstream and downstream to the transcription start site (TSS) and the transcription end site (TES), respectively.

In *A. thaliana*, DNA methylation at transposons and repeats is guided by short interfering RNAs (siRNAs) that recruit the DNA cytosine MTase, Domains Rrearranged Methyltransferase 2 (DRM2), *via* protein/RNA complexes (47,48). Since the 24nt siRNAs guide where DNA methylation occurs in the plant genome, including methylation in the CpG context, we checked if the CpG methylation patterns from bisulfite sequencing data correlate as expected with the optical data at sites that produce 24nt siRNAs (i.e. sites producing 24nt siRNAs should have lower levels of non-methylated CpG sites compared to surrounding regions). Methylation scores from genome-wide bisulfite sequencing data (Supplementary File 5) and relative intensity values from optical mapping data were calculated 10 kbp upstream and downstream relative to the 24nt siRNA clusters (position 0) (39). These data were combined into a single plot representing the methylation profile around these regions (Figure 5A). The methylation score from bisulfite sequencing data (blue) shows a sharp dip around position 0, indicating highly methylated DNA around 24nt siRNA clusters. The relative intensity values from the optical mapping data (red) correlate well with this trend, although at a lower resolution, as expected from the optical diffraction limit.

As negative controls, we examined optical mapping data over primary miRNA loci and several trans-acting siRNA loci (TAS loci) which are not related to DNA methylation (Supplementary Figure S10, Supplementary File 5). As expected, the intensity profile across the miRNA precursor and TAS loci did not have a specific pattern around the cluster center.

In addition to DNA methylation in all sequence contexts at transposons and repeats, some gene bodies are methylated specifically in CpG context in the *A. thaliana* genome and this methylation is distributed away from the 5′ region of genes (11). Thus, we also compared the methylation profiles across gene bodies from genome-wide bisulfite sequencing and optical mapping (Supplementary File 6). Figure 5B illustrates the profiles across the gene bodies and 1.5 kb upstream of the transcription start site (TSS) and downstream from the transcription end site (TES). Again, the two datasets show the expected modulation in methylation along the gene, with sequencing data displaying higher resolution (as seen downstream of the TES). While lacking in resolution, optical mapping has the advantage of visualizing intact genes as well as extremely long DNA molecules that are able to capture both large-scale methylation patterns such as promoters and their distant enhancers (26), and methylation across highly variable regions that are difficult to map by short-read sequencing such as macrostelite arrays (23).

## CONCLUSIONS

In this study, we use the DNA methyltransferase eM.SssI and a synthetic cofactor together with the nucleosidase MTAN to fluorescently label non-methylated CpG dinucleotides. The addition of MTAN to the reaction significantly increased the labeling efficiency of eM.SssI, making the method compatible with single-molecule analysis. Traditional bisulfite sequencing experiments require sampling a CpG site at least 30 times to accurately report on its methylation status. In order to reproduce such methylation calls by single molecule mapping, each CpG must be assessed on at leased 30 individual molecules. With a given experimental coverage of about 50× with optical mapping, we require a minimum labeling efficiency of ∼60% to accurately call methylation status. Thus, the labeling efficiency without MTAN was measured to be too low for an accurate methylation call in genomic DNA in a single experiment, while the increased efficiency with the addition of MTAN is sufficient. Application of this labeling approach to optical genome mapping enables genome-wide methylome profiling, offering long-range information that can span long variable regions and repeats (22,23). We note that this concept may be similarly utilized to map other genomic features such as other epigenetic modifications and DNA damage lesions (22,23,49–52).

Global methylation changes can be quantified by integrating the optical methylation signal for a given amount of DNA (Figure 2). Assessment of whole genome methylation levels may serve as a diagnostic tool that can distinguish between patients with healthy or malignant cells. It is long known that patients with B-cell chronic lymphocytic leukemia (CLL) experience hypermethylation in specific genes (53) while their global methylation levels decrease (45). This hypomethylation can be detected by the presented optical labeling scheme with very high sensitivity, emphasized by the distinct differences in labeling between the healthy and CLL patient (Figure 2B).

The generation of whole-genome methylation maps by means of optical mapping was demonstrated here on the model plant *A. thaliana* and found to correlate well with genome-wide bisulfite sequencing data. As many plant genomes are highly repetitive, optical mapping is becoming a routine method for genomic characterization in plants (54–56). By utilizing this new approach, it is now possible to access methylation information in repetitive or otherwise challenging regions. While optical mapping resolution is limited by the optical diffraction limit to around 1 kb, we increased the effective resolving power of the technique by using fluorescence intensity in addition to genomic location. Thus, CpG-rich regions, where more than one CpG site resides within the optical spot, can now be characterized by signal intensity that reports on labeling density, i.e., the actual number of labeled CpG sites at a given fluorescent spot. Future extension of the method to the human genome may facilitate research on long range epigenetic regulation and for early diagnosis of malignant transformations related to DNA methylation.

## DATA AVAILABILITY

Optical mapping data can be downloaded from the following links: https://submit.ncbi.nlm.nih.gov/ft/byid/mysn1327/moleculequalityreport_q60.cmap_v2.cmap https://submit.ncbi.nlm.nih.gov/ft/byid/vep0xuri/moleculequalityreport.confidence.60.xmap.

## Supplementary Material

gkac460_Supplemental_Files

## References

[1] Schumacher A. , Kapranov P., Kaminsky Z., Flanagan J., Assadzadeh A., Yau P., Virtanen C., Winegarden N., Cheng J., Gingeras T. et al. Microarray-based DNA methylation profiling: Technology and applications. Nucleic Acids Res. 2006; 34:528–542. 16428248 10.1093/nar/gkj461 PMC1345696

[2] Bird A. DNA methylation patterns and epigenetic memory. Genes Dev. 2002; 16:6–21. 11782440 10.1101/gad.947102

[3] Ehrlich M. , Gama-Sosa M.A., Huang L.H., Midgett R.M., Kuo K.C., Mccune R.A., Gehrke C. Amount and distribution of 5-methylcytosine in human DNA from different types of tissues or cells. Nucleic Acids Res. 1982; 10:2709–2721. 7079182 10.1093/nar/10.8.2709 PMC320645

[4] Esteller M. CpG island hypermethylation and tumor suppressor genes: A booming present, a brighter future. Oncogene. 2002; 21:5427–5440. 12154405 10.1038/sj.onc.1205600

[5] Molloy P.L. DNA hypomethylation in cancer. Cancer Epigenet. 2008; 1:7–37.

[6] Ehrlich M. DNA methylation in cancer: Too much, but also too little. Oncogene. 2002; 21:5400–5413. 12154403 10.1038/sj.onc.1205651

[7] Brothman A.R. , Swanson G., Maxwell T.M., Cui J., Murphy K.J., Herrick J., Speights V.O., Isaac J., Rohr L.R. Global hypomethylation is common in prostate cancer cells: A quantitative predictor for clinical outcome. Cancer Genet. Cytogenet. 2005; 156:31–36. 15588853 10.1016/j.cancergencyto.2004.04.004

[8] Seifert H.H. , Schmiemann V., Mueller M., Kazimirek M., Onofre F., Neuhausen A., Florl A.R., Ackermann R., Boecking A., Schulz W.A. et al. In situ detection of global DNA hypomethylation in exfoliative urine cytology of patients with suspected bladder cancer. Exp. Mol. Pathol. 2007; 82:292–297. 17026997 10.1016/j.yexmp.2006.08.002

[9] CATONI G.L. S-Adenosylmethionine; a new intermediate formed enzymatically from L-methionine and adenosinetriphosphate. J. Biol. Chem. 1953; 204:403–416. 13084611

[10] Zhang H. , Lang Z., Zhu J.K. Dynamics and function of DNA methylation in plants. Nat. Rev. Mol. Cell Biol. 2018; 19:489–506. 29784956 10.1038/s41580-018-0016-z

[11] Cokus S.J. , Feng S., Zhang X., Chen Z., Merriman B., Haudenschild C.D., Pradhan S., Nelson S.F., Pellegrini M., Jacobsen S.E. Shotgun bisulphite sequencing of the Arabidopsis genome reveals DNA methylation patterning. Nature. 2008; 452:215–219. 18278030 10.1038/nature06745 PMC2377394

[12] Lister R. , O’Malley R.C., Tonti-Filippini J., Gregory B.D., Berry C.C., Millar A.H., Ecker J.R. Highly integrated single-base resolution maps of the epigenome in Arabidopsis. Cell. 2008; 133:523–536. 18423832 10.1016/j.cell.2008.03.029 PMC2723732

[13] Li P. , Demirci F., Mahalingam G., Demirci C., Nakano M., Meyers B.C. An integrated workflow for DNA methylation analysis. J. Genet. Genomics. 2013; 40:249–260. 23706300 10.1016/j.jgg.2013.03.010

[14] Lorthongpanich C. , Cheow L.F., Balu S., Quake S.R., Knowles B.B., Burkholder W.F. Single-cell DNA-methylation analysis preimplantation embryos. Science. 2013; 341:1110–1112. 24009393 10.1126/science.1240617

[15] Mooijman D. , Dey S.S., Boisset J.C., Crosetto N., Van Oudenaarden A. Single-cell 5hmC sequencing reveals chromosome-wide cell-to-cell variability and enables lineage reconstruction. Nat. Biotechnol. 2016; 34:852–856. 27347753 10.1038/nbt.3598

[16] Song C.X. , Clark T.A., Lu X.Y., Kislyuk A., Dai Q., Turner S.W., He C., Korlach J. Sensitive and specific single-molecule sequencing of 5- hydroxymethylcytosine. Nat. Methods. 2012; 9:75–77. 10.1038/nmeth.1779 PMC3646335 22101853

[17] Flusberg B.A. , Webster D.R., Lee J.H., Travers K.J., Olivares E.C., Clark T.A., Korlach J., Turner S.W. Direct detection of DNA methylation during single-molecule, real-time sequencing. Nat. Methods. 2010; 7:461–465. 20453866 10.1038/nmeth.1459 PMC2879396

[18] Rand A.C. , Jain M., Eizenga J.M., Musselman-Brown A., Olsen H.E., Akeson M., Paten B. Mapping DNA methylation with high-throughput nanopore sequencing. Nat. Methods. 2017; 14:411–413. 28218897 10.1038/nmeth.4189 PMC5704956

[19] Simpson J.T. , Workman R.E., Zuzarte P.C., David M., Dursi L.J., Timp W. Detecting DNA cytosine methylation using nanopore sequencing. Nat. Methods. 2017; 14:407–410. 28218898 10.1038/nmeth.4184

[20] Stoiber M. , Quick J., Egan R., Eun Lee J., Celniker S., Neely R., Loman N., Pennacchio L., Brown J. De novo identification of DNA modifications enabled by genome-guided nanopore signal processing. 2017; bioRxiv doi:10 April 2017, preprint: not peer reviewed 10.1101/094672.

[21] van Dijk E.L. , Jaszczyszyn Y., Naquin D., Thermes C. The Third Revolution in Sequencing Technology. Trends Genet. 2018; 34:666–681. 29941292 10.1016/j.tig.2018.05.008

[22] Gabrieli T. , Sharim H., Nifker G., Jeffet J., Shahal T., Arielly R., Levi-Sakin M., Hoch L., Arbib N., Michaeli Y. et al. Epigenetic optical mapping of 5-hydroxymethylcytosine in nanochannel arrays. ACS Nano. 2018; 12:7148–7158. 29924591 10.1021/acsnano.8b03023 PMC6114841

[23] Sharim H. , Grunwald A., Gabrieli T., Michaeli Y., Margalit S., Torchinsky D., Arielly R., Nifker G., Juhasz M., Gularek F. et al. Long-read single-molecule maps of the functional methylome. Genome Res. 2019; 29:646–656. 30846530 10.1101/gr.240739.118 PMC6442387

[24] Levy-Sakin M. , Grunwald A., Kim S., Gassman N.R., Gottfried A., Antelman J., Kim Y., Ho S.O., Samuel R., Michalet X. et al. Toward single-molecule optical mapping of the epigenome. ACS Nano. 2014; 8:14–26. 24328256 10.1021/nn4050694 PMC4022788

[25] Chaisson M.J.P. , Sanders A.D., Zhao X., Malhotra A., Porubsky D., Rausch T., Gardner E.J., Rodriguez O.L., Guo L., Collins R.L. et al. Multi-platform discovery of haplotype-resolved structural variation in human genomes. Nat. Commun. 2019; 10:1784. 30992455 10.1038/s41467-018-08148-z PMC6467913

[26] Margalit S. , Abramson Y., Sharim H., Manber Z., Bhattacharya S., Chen Y.W., Vilain E., Barseghyan H., Elkon R., Sharan R. et al. Long reads capture simultaneous enhancer–promoter methylation status for cell-type deconvolution. Bioinformatics. 2021; 37:I327–I333. 34252972 10.1093/bioinformatics/btab306 PMC8275347

[27] Renbaum P. , Razin A. Mode of action of the Spiroplasma CpG methylase M.SssI. FEBS Lett. 1992; 313:243–247. 1446743 10.1016/0014-5793(92)81201-v

[28] Renbaum P. , Abrahamove D., Fainsod A., Wilson G.G., Rottem S., Razin A. Cloning, characterization, and expression in *Escherichia coli* of the gene coding for the CpG DNA methylase from *Spiroplasma* sp. strain MQ1(M Sssl). Nucleic Acids Res. 1990; 18:1145–1152. 2181400 10.1093/nar/18.5.1145 PMC330428

[29] Kriukiene E. , Labrie V., Khare T., Urbanavičiute G., Lapinaite A., Koncevičius K., Li D., Wang T., Pai S., Ptak C. et al. DNA unmethylome profiling by covalent capture of CpG sites. Nat. Commun. 2013; 4:2190. 23877302 10.1038/ncomms3190

[30] Borchardt R.T. , Wu Y.S. Potential inhibitors of S-adenosylmethionine-dependent methyltransferases. 1. Modification of the amino acid portion of S-adenosylhomocysteine. J. Med. Chem. 1974; 17:862–868. 4845379 10.1021/jm00254a016

[31] Lukinavičius G. , Tomkuvienè M., Masevičius V., Klimašauskas S. Enhanced chemical stability of AdoMet analogues for improved methyltransferase-directed labeling of DNA. ACS Chem. Biol. 2013; 8:1134–1139. 23557731 10.1021/cb300669x

[32] Cannon L.M. , Butler F.N., Wan W., Sunny Zhou Z. A stereospecific colorimetric assay for (S,S)-adenosylmethionine quantification based on thiopurine methyltransferase-catalyzed thiol methylation. Anal. Biochem. 2002; 308:358–363. 12419350 10.1016/s0003-2697(02)00267-1

[33] Lee B.W.K. , Sun H.G., Zang T., Ju-Kim B., Alfaro J.F., Zhou Z.S. Enzyme-catalyzed transfer of a ketone group from an S-adenosylmethionine analogue: A tool for the functional analysis of methyltransferases. J. Am. Chem. Soc. 2010; 132:3642–3643. 20196537 10.1021/ja908995p PMC2849307

[34] Margalit S. , Avraham S., Shahal T., Michaeli Y., Gilat N., Magod P., Caspi M., Loewenstein S., Lahat G., Friedmann-Morvinski D. et al. 5-Hydroxymethylcytosine as a clinical biomarker: fluorescence-based assay for high-throughput epigenetic quantification in human tissues. Int. J. Cancer. 2020; 146:115–122. 31211411 10.1002/ijc.32519

[35] Jain N. , Shahal T., Gabrieli T., Gilat N., Torchinsky D., Michaeli Y., Vogel V., Ebenstein Y. Global modulation in DNA epigenetics during pro-inflammatory macrophage activation. Epigenetics. 2019; 14:1183–1193. 31262215 10.1080/15592294.2019.1638700 PMC6791700

[36] Torchinsky D. , Ebenstein Y. Sizing femtogram amounts of dsDNA by single-molecule counting. Nucleic Acids Res. 2016; 44:e17. 26365235 10.1093/nar/gkv904 PMC4737178

[37] Arielly R. , Ebenstein Y. Irys Extract. Bioinformatics. 2018; 34:134–136. 29036307 10.1093/bioinformatics/btx437 PMC5870776

[38] Kawakatsu T. , Huang S. shan C., Jupe F., Sasaki E., Schmitz R.J.J., Urich M.A.A., Castanon R., Nery J.R.R., Barragan C., He Y. et al. Epigenomic diversity in a global collection of *Arabidopsis thaliana* accessions. Cell. 2016; 166:492–505. 27419873 10.1016/j.cell.2016.06.044 PMC5172462

[39] Zhou M. , Palanca A.M.S., Law J.A. Locus-specific control of the de novo DNA methylation pathway in Arabidopsis by the CLASSY family. Nat. Genet. 2018; 50:865–873. 29736015 10.1038/s41588-018-0115-y PMC6317521

[40] Zhang C. , Li G., Zhu S., Zhang S., Fang J. TasiRNAdb: a database of ta-siRNA regulatory pathways. Bioinformatics. 2014; 30:1045–1046. 24371150 10.1093/bioinformatics/btt746

[41] Quinlan A.R. , Hall I.M. BEDTools: a flexible suite of utilities for comparing genomic features. Bioinformatics. 2010; 26:841–842. 20110278 10.1093/bioinformatics/btq033 PMC2832824

[42] Ramírez F. , Ryan D.P., Grüning B., Bhardwaj V., Kilpert F., Richter A.S., Heyne S., Dündar F., Manke T. deepTools2: a next generation web server for deep-sequencing data analysis. Nucleic Acids Res. 2016; 44:W160–W165. 27079975 10.1093/nar/gkw257 PMC4987876

[43] Ronning D.R. , Iacopelli N.M., Mishra V. Enzyme-ligand interactions that drive active site rearrangements in the Helicobacter pylori 5′-methylthioadenosine/S-adenosylhomocysteine nucleosidase. Protein Sci. 2010; 19:2498–2510. 20954236 10.1002/pro.524 PMC3009416

[44] Baylin S.B. , Jones P.A. A decade of exploring the cancer epigenome-biological and translational implications. Nat. Rev. Cancer. 2011; 11:726–734. 21941284 10.1038/nrc3130 PMC3307543

[45] Wahlfors J. , Hiltunen H., Heinonen K., Hamalainen E., Alhonen L., Janne J. Genomic hypomethylation in human chronic lymphocytic leukemia. Blood. 1992; 80:2074–2080. 1382719

[46] Matzke M.A. , Mosher R.A. RNA-directed DNA methylation: an epigenetic pathway of increasing complexity. Nat. Rev. Genet. 2014; 15:394–408. 24805120 10.1038/nrg3683

[47] Law J.A. , Du J., Hale C.J., Feng S., Krajewski K., Palanca A.M.S., Strahl B.D., Patel D.J., Jacobsen S.E. Polymerase IV occupancy at RNA-directed DNA methylation sites requires SHH1. Nature. 2013; 498:385–389. 23636332 10.1038/nature12178 PMC4119789

[48] Zhao Y. , Chen X. Non-coding RNAs and DNA methylation in plants. Natl. Sci. Rev. 2014; 1:219–229. 25635229 10.1093/nsr/nwu003 PMC4307843

[49] Michaeli Y. , Shahal T., Torchinsky D., Grunwald A., Hoch R., Ebenstein Y. Optical detection of epigenetic marks: sensitive quantification and direct imaging of individual hydroxymethylcytosine bases. Chem. Commun. 2013; 49:8599–8601. 10.1039/c3cc42543f 23756466

[50] Torchinsky D. , Michaeli Y., Gassman N.R., Ebenstein Y. Simultaneous detection of multiple DNA damage types by multi-colour fluorescent labelling. Chem. Commun. 2019; 55:11414–11417. 10.1039/c9cc05198h PMC6783632 31482872

[51] Zirkin S. , Fishman S., Sharim H., Michaeli Y., Don J., Ebenstein Y. Lighting up individual DNA damage sites by in vitro repair synthesis. J. Am. Chem. Soc. 2014; 136:7771–7776. 24802414 10.1021/ja503677n

[52] Gilat N. , Tabachnik T., Shwartz A., Shahal T., Torchinsky D., Michaeli Y., Nifker G., Zirkin S., Ebenstein Y. Single-molecule quantification of 5-hydroxymethylcytosine for diagnosis of blood and colon cancers. Clin. Epigenet. 2017; 9:70. 10.1186/s13148-017-0368-9 PMC5512773 28725280

[53] Issa J.P.J. DNA methylation changes in hematologic malignancies: Biologic and clinical implications. Leukemia. 1997; 11:S7–11. 9130685

[54] Deschamps S. , Zhang Y., Llaca V., Ye L., Sanyal A., King M., May G., Lin H. A chromosome-scale assembly of the sorghum genome using nanopore sequencing and optical mapping. Nat. Commun. 2018; 9:4844. 30451840 10.1038/s41467-018-07271-1 PMC6242865

[55] Michael T.P. , Bryant D., Gutierrez R., Borisjuk N., Chu P., Zhang H., Xia J., Zhou J., Peng H., El Baidouri M. et al. Comprehensive definition of genome features in Spirodela polyrhiza by high-depth physical mapping and short-read DNA sequencing strategies. Plant J. 2017; 89:617–635. 27754575 10.1111/tpj.13400

[56] Jiao W.B. , Accinelli G.G., Hartwig B., Kiefer C., Baker D., Severing E., Willing E.M., Piednoel M., Woetzel S., Madrid-Herrero E. et al. Improving and correcting the contiguity of long-read genome assemblies of three plant species using optical mapping and chromosome conformation capture data. Genome Res. 2017; 27:778–786. 28159771 10.1101/gr.213652.116 PMC5411772

