# Supplementary material for: Chemoenzymatic labeling of DNA methylation patterns for single-molecule epigenetic mapping
Source: Nucleic Acids Res. 2022 Jun 3;50(16):e92. doi: 10.1093/nar/gkac460 (PMC9458417; doi:10.1093/nar/gkac460)
Supplement: gkac460_Supplemental_Files [file gkac460_supplemental_files.zip › Final Suporting information file.docx]

**Supporting information**

**Chemoenzymatic labeling of DNA methylation patterns for single-molecule epigenetic mapping**

**Tslil Gabrieli^1^, Yael Michaeli^1^, Sigal Avraham^1^, Dmitry Torchinsky^1^, Sapir Margalit^1^, Leonie Schütz^2^ Matyas Juhasz^2^, Ceyda Coruh^3^, Nissim Arbib^4^, Zhaohui Sunny Zhou^5^, Julie A. Law^3^, Elmar Weinhold*^2^, Yuval Ebenstein*^1^**

^1^ School of Chemistry, Center for Nanoscience and Nanotechnology, Center for Light-Matter Interaction, Raymond and Beverly Sackler Faculty of Exact Sciences, Tel Aviv University, Tel Aviv, Israel.

^2^ Institute of Organic Chemistry, RWTH Aachen University, D-52056 Aachen Germany

^3^ Plant Molecular and Cellular Biology Laboratory, Salk Institute for Biological Studies, La Jolla, CA, USA

^4^ Department of Obstetrics and Gynecology, Meir Hospital, Kfar Saba, Israel & Sackler Faculty of Medicine, Tel Aviv University, Tel Aviv, Israel.

^5^ Department of Chemistry and Chemical Biology, and Barnett Institute of Chemical and Biological Analysis, Northeastern University, Boston, Massachusetts 02115, USA

**Figure S1.** Quantification of non-methylated cytosine on labeled DNA molecules.

**
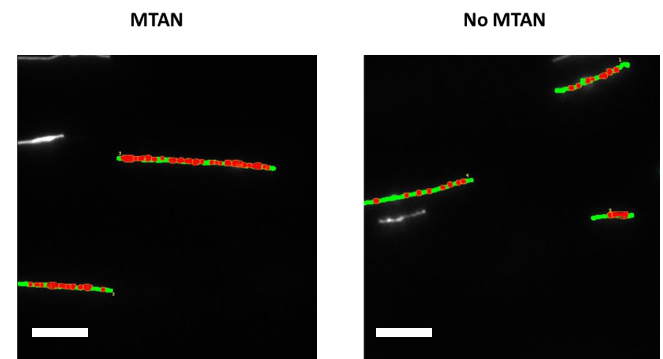
**

Representative image of unmethylated λ DNA stretched on activated glass slides and analyzed by an in-house developed software for measuring intensity profiles of colocalized non-methylated cytosine labels and stained DNA molecules. In green, YOYO-1 labeled molecules that were detected by the software. In gray, DNA molecules labeled with YOYO-1 that were rejected from the analysis due to non-linear stretching or crossing with another molecule. Labels at non-methylation CpG sites that were detected by the software are shown in red. Scale bars correspond to 20 kbp.

**Figure S2.** Labeling intensity profile measurement of eM.SssI, with or without MTAN.


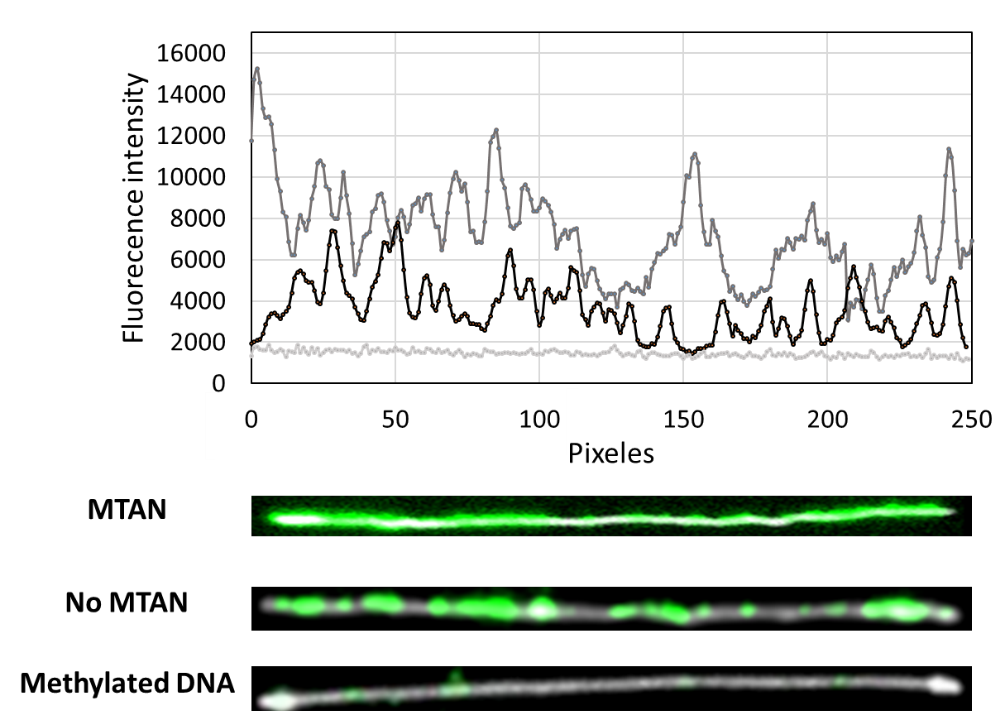

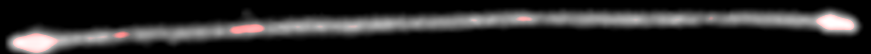

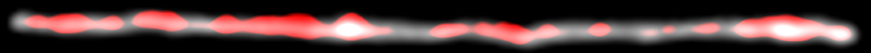

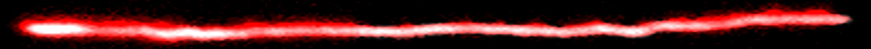

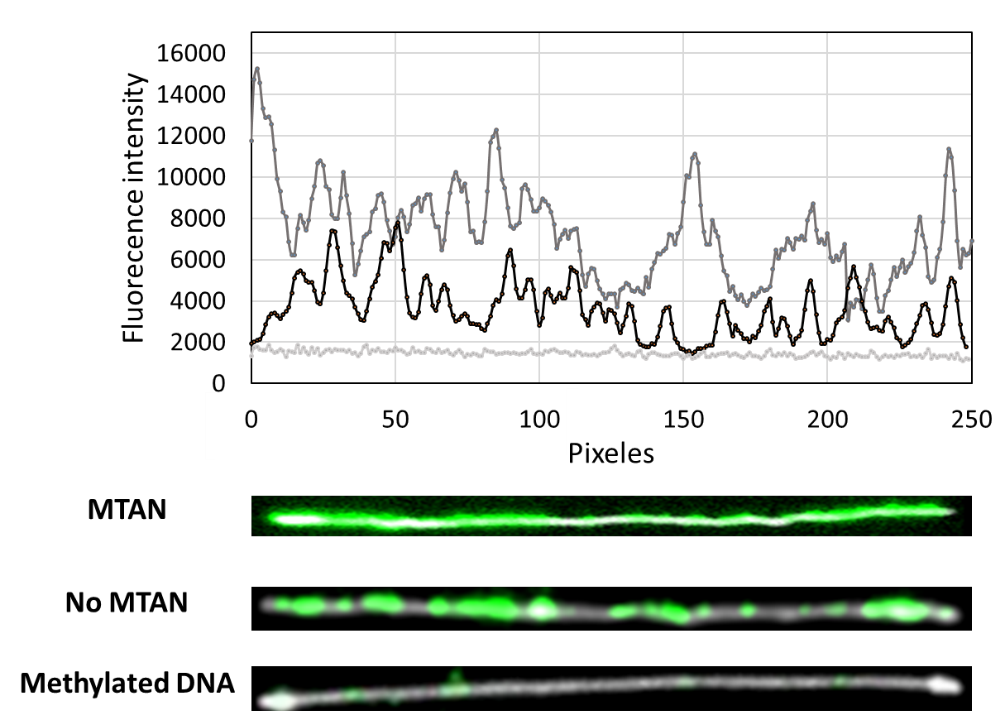


Top panel: Fluorescence intensity profile across unmethylated λ DNA, labeled with eM.SssI at CpG sites, with (dark gray) or without MTAN (black). Methylated λ was used as control (light gray). Bottom panel: Representative images of λ DNA molecules labeled with eM.SssI in the presence of MTAN on unmethylated DNA, in the absence of MTAN on unmethylated DNA and on methylated DNA. Here the DNA backbone is shown in gray and the labels marking non-methylated CpG sites are shown in red. Red images intensities were scaled for visualization.

**Figure S3.** Relative efficiency measurements of non-methylation labeling.

**
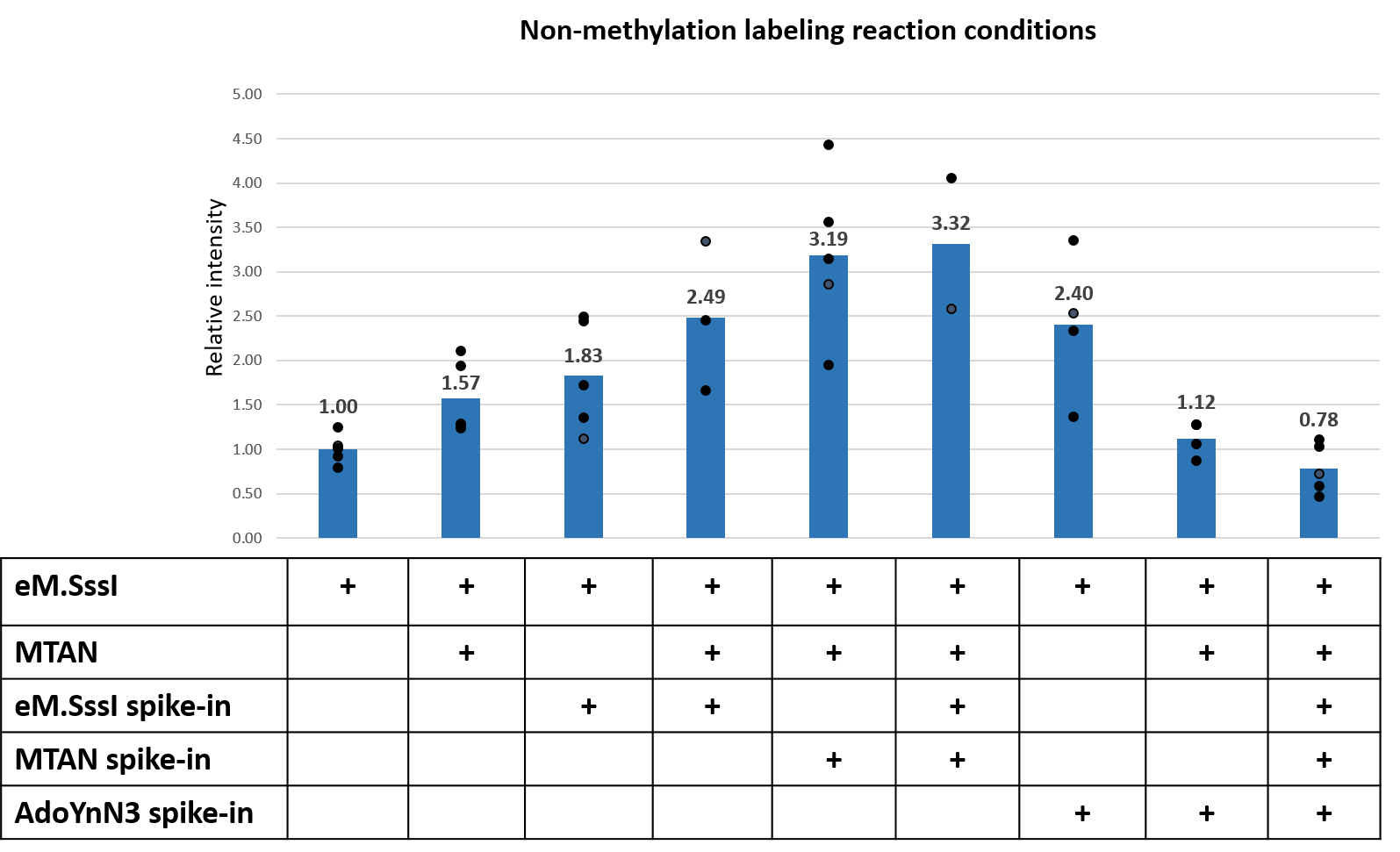
**

Non-methylation labeling reaction was performed under different condition for optimization. Where mentioned, a spike-in was performed after two hours of incubation. Samples were then loaded on a multi-well slide for relative intensity measurement. 2-5 replicates were conducted for each condition. Bar graph represent the mean methylation value and black circles are the results of the replicates. Best results were obtained when both eM.SssI and MTAN were spiked in. These conditions were further used for optical mapping experiments.

**Figure S4**. Modification-restriction assays to analyze the activity of eM.SssI and AdoYnAzide in the absence and presence of MTAN.

**
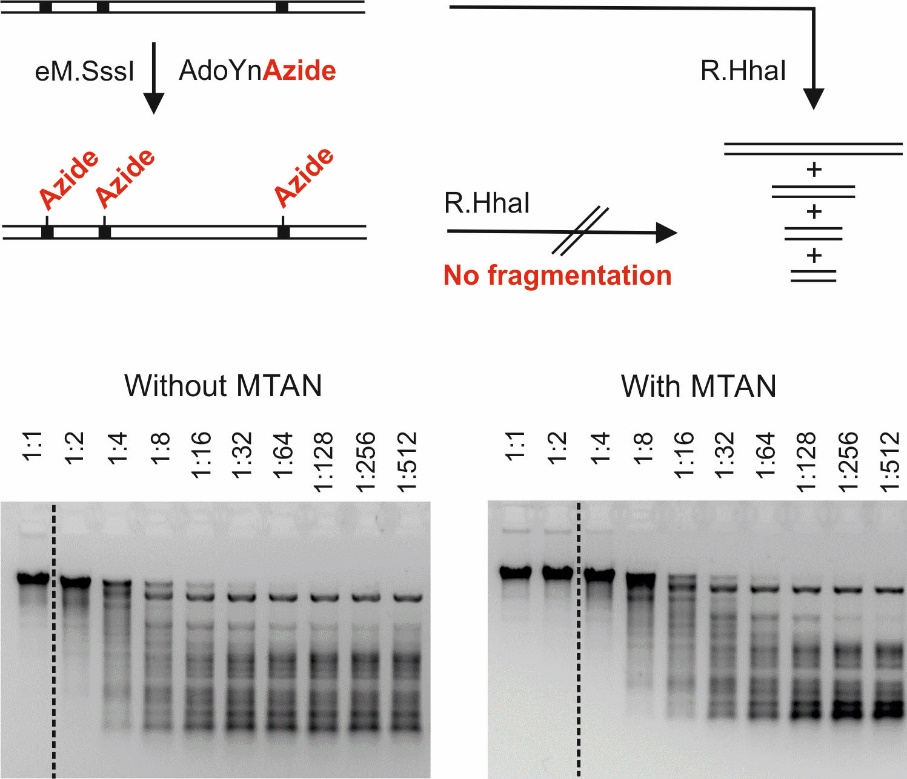
**

Top panel: Schematic representation of the modification-restriction assay. DNA is incubated with eM.SssI and cofactor AdoYnAzide, challenged with the modification-sensitive restriction endonuclease R.HhaI (5’-G**CG**C-3’) and fragmentation analyzed by agarose gel electrophoresis. Completely alkylated DNA is fully protected against fragmentation by R.HhaI while incomplete or no alkylation leads to partial or full DNA fragmentation.

Bottom panel: Activity of eM.SssI without (left) and with (right) MTAN. Twofold serial eM.SssI dilutions starting with 1 equivalent per CpG site are incubated with AdoYnAzide and λ DNA at 37 °C for 1 h, supplemented with R.HhaI and fragmentation is analyzed by agarose gel (1%) electrophoresis. The lowest ratios of enzyme to recognition sites needed to fully protect λ DNA are indicated by dashed lines. Activity of eM.SssI is doubled in the presence of MTAN under these conditions.

A full description of the experimental procedure is given in the materials and methods section.

**Figure S5.** HPLC efficiency assay for quantification of nucleosides obtained in the absence and presence of MTAN. See a full description of the experimental procedure in the materials and methods section.

**A.**

**
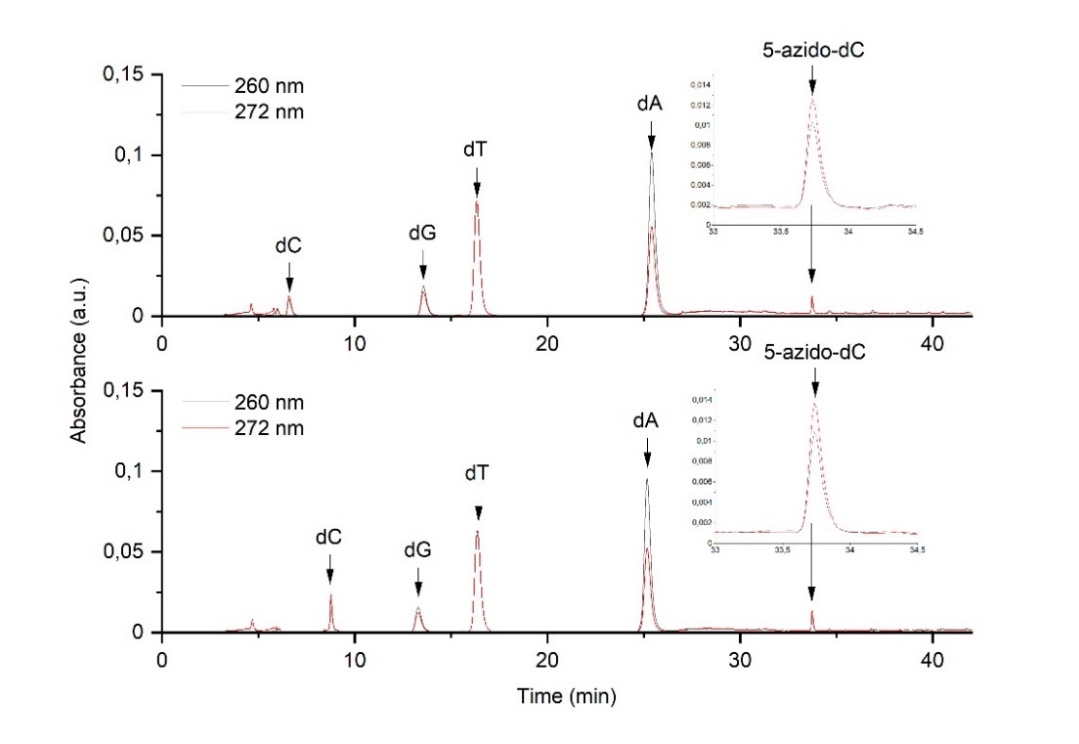
**

**B.**

**
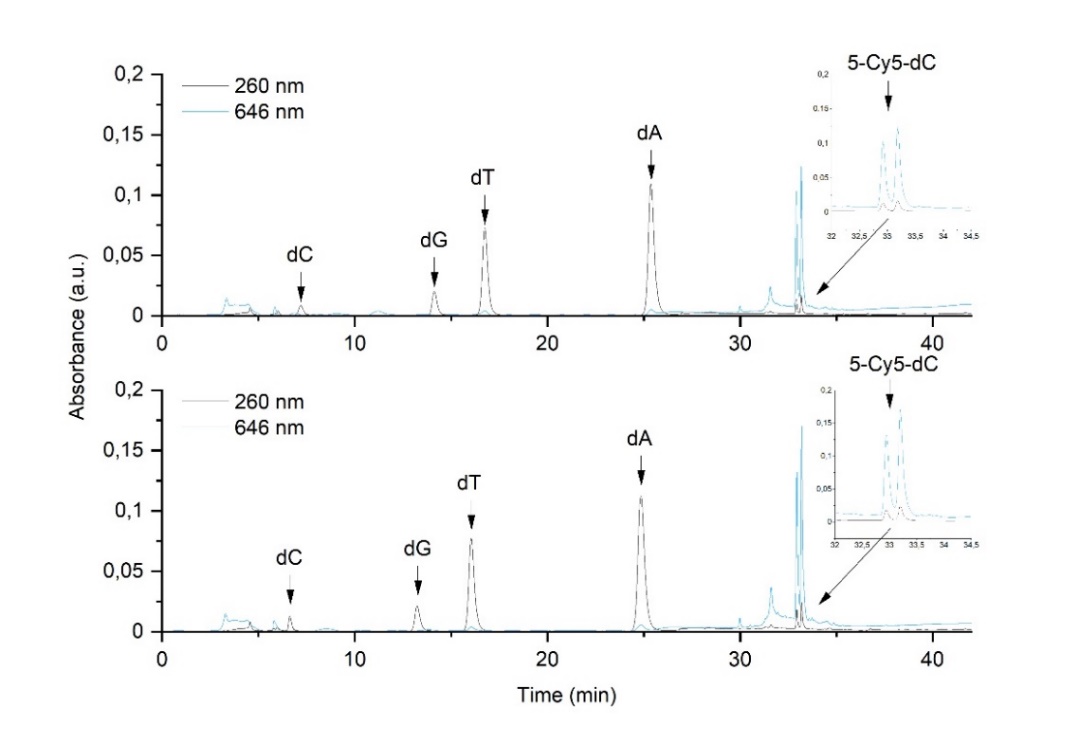
**

A: Reverse-phase HPLC analysis of nucleosides obtained from modification of a duplex oligodeoxynucleotide (ODN) with eM.SssI and cofactor AdoYnAzide (1. step) in the absence (upper) and presence (lower) of MTAN followed by enzymatic fragmentation.

B: Same as above but with additional fluorescence labeling using DBCO-Sulfo-Cy5 (2. step) before enzymatic fragmentation. An increased signal for the fluorescently labeled nucleoside 5-Cy5-dC is clearly visible for incubation with MTAN (lower) compared to incubation without MTAN (upper) in the 1. step.

**Table S1:** Quantification of nucleosides obtained in the absence and presence of MTAN for enzymatic transfer of azide groups (1. step) and fluorescence labeling (2. step) of a duplex ODN.

|  | dC | dG | dT | dA | 5-azido-dC | 5-Cy5-dC |
| --- | --- | --- | --- | --- | --- | --- |
| Duplex ODN  1. Step No MTAN With MTAN  2. Step No MTAN With MTAN | 4  2.50 2.46  2.63 2.27 | 4  4.00 4.02  3.96 3.92 | 20  20.00 20.00  20.00 20.00 | 20  18.68 18.97  18.77 19.10 | 1.26 (67%) 1.63 (80%)  0.00 0.00 | 1.26 (100%) 1.63 (100%) |

To calculate labeling efficiency, peaks of the nucleosides at 260 nm were integrated using Empower 2 software and areas were normalized by dividing them by the respective extinction coefficients at 260 nm (dC: 7009 mol l-1 cm-1; dG: 11715 mol l-1 cm-1; dT: 8902 mol l-1 cm-1; dA: 15663 mol l-1 cm-1; 5-azido-dC = 5mdC: 5435 mol l-1 cm-1). Normalized areas of dT were divided by the number of dT residues in the duplex ODN (20 residues) to obtain a normalized area for one nucleoside. Normalized areas for the other nucleosides were divided by this value to give experimental amounts for all nucleosides. Yields for 5-azido-dC were calculated by dividing the amount of 5-azido-dC by the sum of dC and 5-azido-dC multiplied by 2 (four dC/two target dC). Yields for 5-Cy5-dC were calculated from the decrease of 5-azido-dC.

**Figure S6.** Linear dependency between the number of CpG sites and fluorescent signal.

**
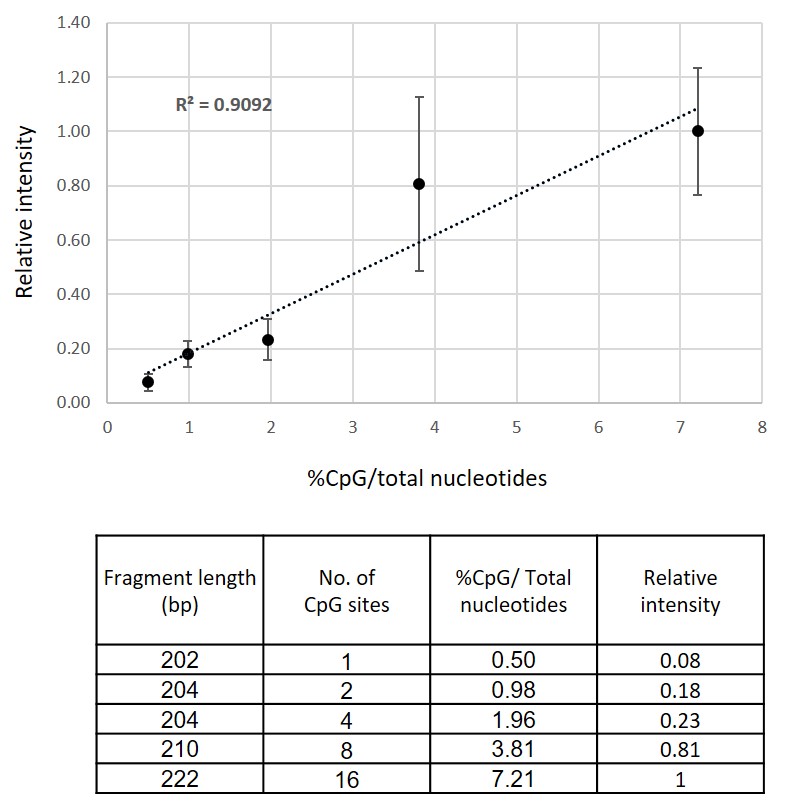
**

To demonstrate the linear dependency between the number of CpG sites and the fluorescent signal, we generated 5 PCR products with a similar length that contain a different number of CpG sites. The products were labeled as described (2-5 replicates for each PCR product) and analyzed by a multi-well slide assay. Relative intensity for each PCR product was plotted as a function of %CpG sites. To calculate %CpG sites, the number of CpGs in a given fragment was divided by the number of base pairs composing the fragment. Relative intensity values were obtained by dividing the fluorescent signal from labeled CpG sites by the DNA stain signal (total DNA) following subtraction of the background signal. Results show a linear increase in the fluorescent signal with increasing the number of CpG sites.

**
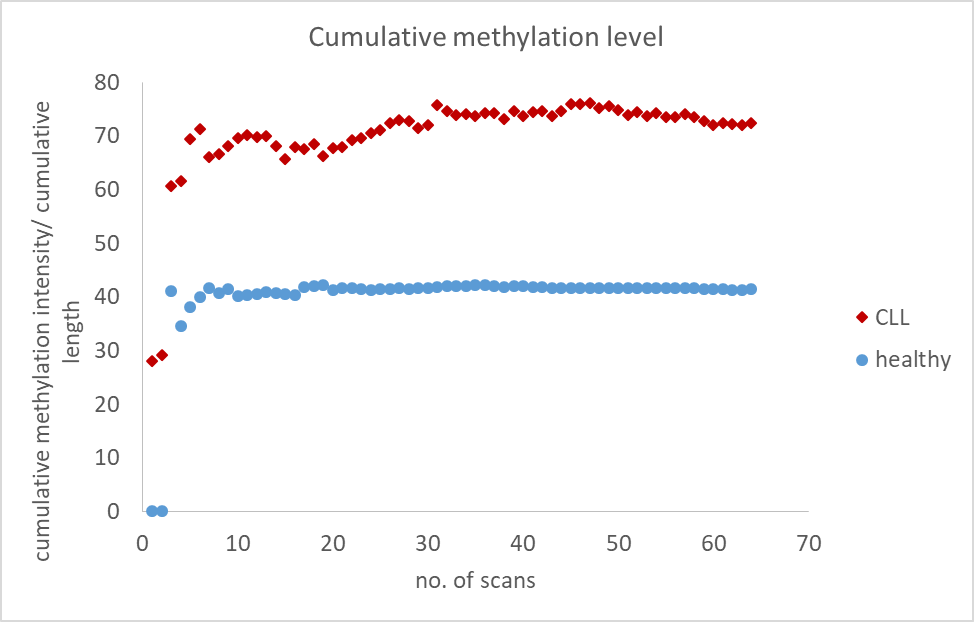
Figure S7.** Cumulative methylation label density as a function of a number of scans.

To verify that a sufficient amount of data was collected, the accumulated running average of randomly chosen scans was calculated. As observed, the average stabilizes after 5-10 scans, indicating that the amount of data analyzed is sufficient even when sampling only 10% of our data.

We note that despite sampling ~17 times less DNA for the healthy sample, this has no effect on the reliability of the results since such large amounts of data are not needed for a reliable assessment of global methylation levels.

**Figure S8.** Representative *in silico* representation of molecules aligned to chromosome 3.


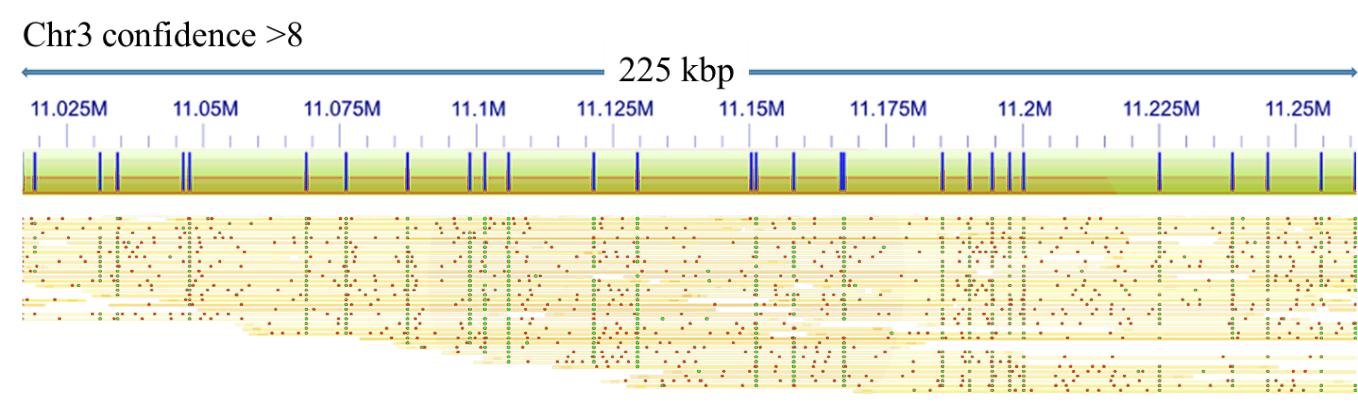


In green, genetic labels using Nt. BspQI, and in red, labels for non-methylated CpG sites marked by eM.SssI.

**Figure S9.** Optical methylation profile of a representative imaged DNA molecule in comparison with locations of CpG sites.

**
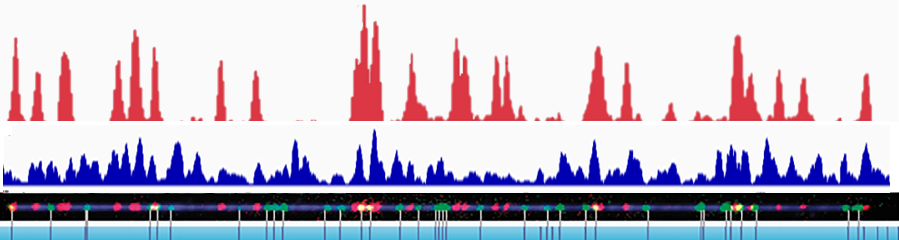
**

A 150 kbp DNA molecule is aligned to chromosome 3 by the green genetic labels. The vertical lines on the blue strip represent the theoretical positions of genetic labels in the reference genome. The fluorescence intensity pattern representing the levels of non-methylated CpG sites along the molecule is presented in red. CpG sites extracted from TAIR10 genome assembly of *A. thaliana* are presented in the blue track. Both tracks are showing the levels of non-methylated CpG sites.

**Figure S10.** Non-methylated CpG sites over miRNA precursor and TAS loci.

**
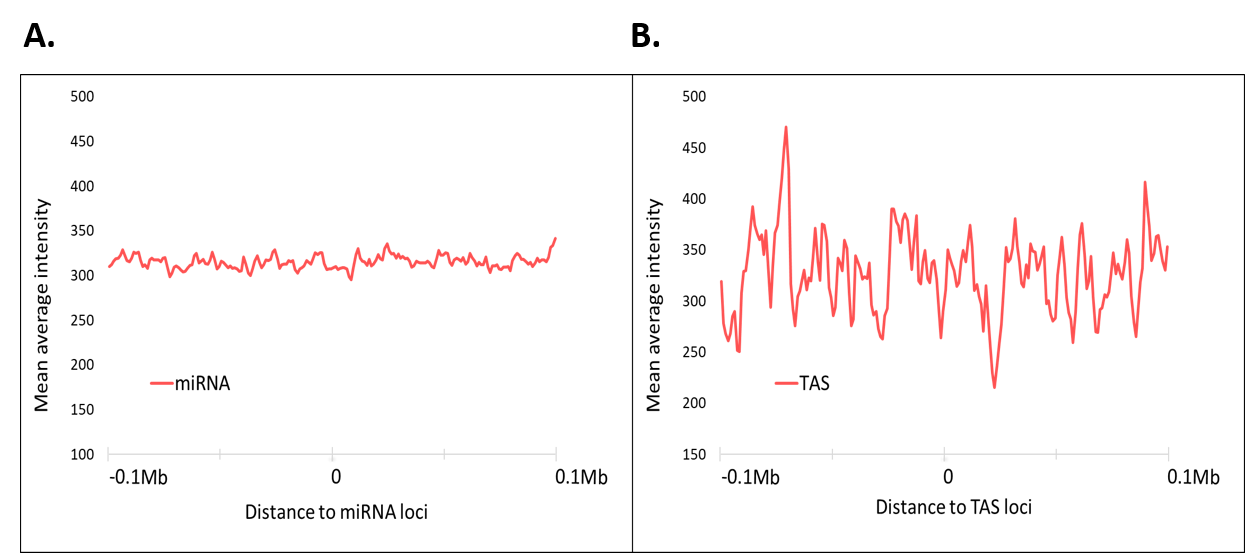
**

Optical mapping average intensity of non-methylated CpG sites across **A**. miRNA precursor loci **B**. TAS loci.
