# Supplementary material for: Chemoenzymatic labeling of DNA methylation patterns for single-molecule epigenetic mapping
Source: Nucleic Acids Res. 2022 Jun 3;50(16):e92. doi: 10.1093/nar/gkac460 (PMC9458417; doi:10.1093/nar/gkac460)
Supplement: gkac460_Supplemental_Files [file gkac460_supplemental_files.zip › Supplementary files.docx]

Supplementary file 1- additional results as described in the main text (PDF)

Supplementary file 2- optical mapping intensity healthy blood sample.

Supplementary file 3- optical mapping intensity CLL sample.

Supplementary file 4- comparison between non- methylation CpG sites in optical mapping vs. bisulfite sequencing.

Supplementary file 5- 24nt siRNA, miRNA precursor loci and TAS loci in *A. thaliana*.

Supplementary file 6- genomic locations of *A. thaliana* genes.
